# Free from conspiracies: The negative relationship between societal freedom and belief in generic and content‐specific conspiracy theories

**DOI:** 10.1111/bjso.70021

**Published:** 2025-11-24

**Authors:** Maciej Siemiątkowski, Theofilos Gkinopoulos, Michał Bilewicz

**Affiliations:** ^1^ Faculty of Psychology University of Warsaw Warsaw Poland; ^2^ Department of Social Sciences, School of Humanities and Social Sciences University of Nicosia Athens Campus Athens Greece; ^3^ Institute of Psychology Jagiellonian University Kraków Poland

**Keywords:** conspiracy theories, freedom, liberty, political anger

## Abstract

Through five studies, this research examined how objectively measured societal freedom and individual perceptions of it are related to reduced belief in conspiracy theories. Study 1 (*N* = 6353 participants from 36 countries) examined the negative relationship between societal freedom (as measured by the Human Freedom Index) and generic conspiracy beliefs. Study 2 (*N* = 44,458 participants from 52 countries) focused on interest group‐related COVID‐19 conspiracy beliefs– a measure not explicitly referring to government actors. Moving to the individual level, Study 3 (*N* = 278) examined relationships between perceived societal freedom and various conspiracy beliefs, while Study 4 (*N* = 246) experimentally tested whether manipulating perceptions of societal freedom affected belief in generic conspiracist beliefs as well as those related to vaccines and financial crises. Results indicated that both greater societal freedom and higher perceived societal freedom are associated with lower levels of conspiracy beliefs. In Study 5 (*N* = 592), we examined the psychological mechanisms mediating the relationship between perceived societal freedom and conspiracy beliefs and found the significant indirect effect via political anger. These findings contribute to a broader understanding of how macro‐level conditions can be incorporated into efforts to reduce the prevalence of conspiracy theories.

In recent decades, psychological research has begun examining how restrictions and perceptions of freedom shape behaviour and belief formation, particularly in response to emerging societal challenges (Szumowska et al., 2024). This research is built upon foundational work in self‐determination theory, which postulates that individuals experience freedom when their actions align with their core values and preferences (Deci & Ryan, 2000). Supporting this theoretical framework, extensive empirical work has established links between freedom of choice and personal control and self‐identity (Iyengar & Lepper, 1999; Kim & Sherman, 2007; Van Prooijen, 2009). Moreover, studies found that when individuals cannot act consistent with their enduring desires, interests, values and preferences, their decision‐making ability is compromised (Lau & Wenzel, 2015) and their psychological well‐being declines (Chen et al., 2015). The psychological consequences of freedom restriction are particularly pronounced during crises and disasters. Cheek et al. (2022) summarize extensive research indicating that perceived excessive or controlling restrictions can trigger psychological reactance, which is an aversive motivational state involving anger, oppositional thoughts and determined efforts to restore freedom (Brehm, 1966; Brehm & Brehm, 1981; Dillard & Shen, 2005). Research on reactance has also found that perceived excessively controlling policies can elicit strong opposing attitudes and paradoxically reinforce the beliefs and behaviours they aim to change (Rains, 2013; Worchel & Brehm, 1970).

Building on the link between freedom restrictions and the adoption of opposing attitudes and beliefs, we propose that such restrictions may also foster conspiracy beliefs. Conspiracy beliefs, defined as explanations for important events that involve secret plots by powerful and malevolent groups (Douglas et al., 2017), have grown in popularity across various segments of society and carry significant implications for social cohesion and democratic processes (Papaioannou et al., 2023). During societal threats or crises, such beliefs can influence reactionary behaviour by altering how people perceive the legitimacy of authorities (Thomas et al., 2024). Moreover, conspiracy theories serve as explanatory narratives that systematically call into question the procedural fairness of authorities and institutions, predicting sustained perceptions of injustice and diminished institutional trust (Frenzel et al., 2025; Mari et al., 2022). These dynamics are likely to be heightened when freedoms are restricted, contributing to a belief system that reacts against governments and other institutions. However, despite their significance, the psychological mechanisms linking freedom experiences to conspiracy belief adoption remain not fully understood. Similarly, research has not focused on how societal‐level freedom shapes individual belief systems. Therefore, the purpose of this research was to explore the relationship between freedom and conspiracy beliefs as well as the psychological mechanisms that mediate this effect.

## METHODOLOGICAL FRAMEWORK: SOCIETAL FREEDOM AND THE TWO LEVELS ON WHICH IT COULD BE CONCEPTUALIZED

Freedom, as discussed in liberal theories and exemplified in the works of Mill (1859/1999), Berlin (1969) and Rawls (1999), has multiple facets—ranging from individual autonomy (freedom as autonomy) to broader civic and political dimensions. Modern societies vary considerably in how they enact and guarantee these freedoms, which has led to different metrics that attempt to capture the complexity of what we call *societal freedom*. Following Naito's (2007) tripartite conceptualization (which includes freedom as autonomy, political freedom, and conditions for substantive freedom), Rawls's (1999) emphasis on socioeconomic resources, as well as the focus on negative freedom (Berlin, 1969) adopted in the Human Freedom Index (HFI; Vásquez & Porčnik, 2019), our approach defines societal freedom as the absence of coercive institutional, legal and structural constraints that would otherwise prevent individuals from making choices and exercising their rights in the country. By conceptualizing freedom at the societal level, we emphasize the structural and institutional underpinnings—such as the rule of law, economic opportunity and the protection of civil liberties—that encompass the freedoms and rights that particular societies possess. This macro‐level view is complemented by the individual‐level lens, which captures the subjective sense of living in a society that provides these freedoms. Together, these two levels provide a more comprehensive framework for understanding a range of psychological outcomes, as research demonstrated that objective measures of societal conditions (including freedom) and their subjective individual perceptions operate in a complementary manner (Scholten et al., 2017). However, the correlation between actual and perceived freedom is only moderate (Brulé & Veenhoven, 2014), which highlights the importance of studying both of them.

### Country‐level (macro‐level) societal freedom

Objectively measured, country‐level societal freedom refers to institutional and structural factors reflecting the absence of coercive constraints, which echoes Berlin's (1969) concept of negative freedom. This concept focuses specifically on the absence of humanly imposed barriers to an individual's potential actions (unlike positive freedom, which is the capacity to free oneself from any constraints, allowing an individual to find his or her true self (McMahon, 2012)). This conceptual foundation makes negative freedom particularly suitable for empirical measurement because it focuses on observable, objective constraints.

Based on this theoretical foundation, the Human Freedom Index (HFI; Vásquez et al., 2023) is one of the most comprehensive attempts to objectively measure societal freedom across countries. The HFI operationalizes Berlin's (1969) concept of negative freedom by systematically measuring the absence of constraints across multiple domains of human activity. It uses third‐party data to evaluate actual restrictions on individual liberty, ensuring replicability across different contexts and time periods. The index systematically captures various factors, including the rule of law, safety and security, freedom of movement, freedom of religion, freedom of association, assembly and civil society, freedom of expression and information, and economic freedom. By combining these measures, the HFI provides a holistic view of a country's overall freedom level (Vásquez & Porčnik, 2019). This approach aligns with liberal theories of freedom, which emphasize institutional design, equitable access to resources and legal frameworks as key components of a free society (Rawls, 1999; Sen, 1999).

### Individual‐level perceptions of societal freedom

While macro‐level indicators such as the HFI provide valuable insights into societal constraints, understanding how individuals perceive their freedom is an equally important dimension of analysis. Studies have shown that individual perceptions of societal conditions may offer a unique insight into psychological outcomes beyond what can be explained by objective measures alone, as demonstrated in research on economic situation or socioeconomic status (Demakakos et al., 2008; Hornsey, Pearson, et al., 2023). We argue that personal perceptions of societal freedom may differ significantly from formal or objective measures. Although macro‐level conditions inherently set certain limits on how individuals experience constraints, the interpretation of these conditions varies considerably across populations. For example, in societies that score high on the HFI, certain individuals or demographic groups may nonetheless experience significant constraints on political engagement, public discourse, or economic opportunities—whether due to prevailing sociocultural norms, perceived discrimination, or resource constraints. Conversely, in societies with lower HFI scores, individuals may experience considerable freedom in certain life domains, influenced by their particular circumstances, community norms, or sense of personal agency (see, for example, Radkiewicz's (2021) work on extrinsic freedom, which is defined as the unrestricted pursuit of freedom in personal goals, even if it is harmful to others. Individuals who hold such beliefs may often perceive social regulations as constraints, even if these regulations serve to protect collective freedoms.)

## HOW SOCIETAL FREEDOM MIGHT BE RELATED TO CONSPIRACY BELIEFS?

Passini (2017) pointed out that when freedom is restricted, people may respond by rationalizing the restrictions or justifying them as necessary for security and maintaining the status quo (Jost, 2011; Laurin et al., 2012). We propose that another response to restrictions on freedom, whether at the country level or subjectively experienced by individuals, may be the formation or reinforcement of conspiracy beliefs, which, in such cases, would serve as an accessible means of protecting one's identity and worldview (Van Prooijen, 2022). This hypothesis is based on several interrelated theoretical frameworks that enabled us to identify two psychological mechanisms underlying the relationship between societal freedom and conspiracy beliefs. First, psychological reactance theory suggests that when freedoms are restricted, individuals experience threats to their autonomy and sense of control (Brehm, 1966; Brehm & Brehm, 1981). Furthermore, Van Prooijen and Acker (2015) showed that conspiracy beliefs serve as a sense‐making tool in contexts of societal threat to control.

Second, perceived restrictions evoke strong emotional responses, with anger playing an important role in how individuals react to limitations on their freedom. Early psychological reactance theory showed that restrictions on freedom can trigger various resistance behaviours, such as counterarguing, disparaging the source, or even aggression. These reactions have been found to be consistently fuelled by feelings of irritation and anger (Brehm, 1966; Brehm & Brehm, 1981). Dillard and Shen (2005) explicitly captured this in their intertwined‐process model, describing reactance as a combination of negative cognitions and an angry emotional component. Supporting this framework, Rains's (2013) meta‐analysis found evidence that anger is a consequence of threats to freedom. Furthermore, research suggests that anger plays an important role in conspiracy belief endorsement, with trait anger being positively associated with specific and generic conspiracy beliefs (Harmon‐Jones & Szymaniak, 2023; Szymaniak et al., 2023).

We propose that conspiracy beliefs are influenced by low and high societal freedom through the same psychological mechanisms, which are working in opposite directions. When societal freedom is restricted, individuals experience heightened anger, especially regarding the political domain from which the restrictions originate, as well as reduced personal control. These psychological states create a need for explanatory frameworks that restore meaning, making conspiracy theories a particularly appealing form of explanation (see Van Prooijen, 2022). Conversely, when societal freedom is high, political anger is reduced and perceived control increases, thereby decreasing the appeal and need for conspiratorial explanations.

When examining the relationship between societal freedom and conspiracy beliefs, it is important to consider that conspiratorial thinking may reflect real signals arising from events within society. Studies have shown that, in highly corrupt contexts where conspiracies and secret alliances are common, conspiracy beliefs can develop in response to these environmental cues (Alper, 2023; Alper & Imhoff, 2023). Therefore, to comprehensively examine the relationship between societal freedom and conspiracy beliefs, our research employed multiple constructs of conspiracy beliefs that capture distinct aspects of this phenomenon. Generic conspiracy beliefs capture a more general distrust of official narratives and authorities (Kay & Slovic, 2023; Lantian et al., 2016) and address the aspect of conspiracy belief formation related to the detection of cues (Alper, 2023) under freedom restrictions. Conversely, the conspiracy mentality represents a generalized conspiratorial worldview and a readiness to interpret world events as being caused by secret plots (Imhoff & Bruder, 2014). Content‐specific conspiracy beliefs, such as those concerning vaccines (Toribio‐Flórez et al., 2025) or financial crises (van Prooijen et al., 2015), reflect the adaptation of conspiratorial thinking to specific contexts. Notably, vaccine‐related conspiracy beliefs focus on scientific misconduct rather than government control or freedom restrictions. Thereby, this measure extends beyond direct government‐related concerns to areas where conspiratorial thinking may manifest independently of political distrust.

## OVERVIEW OF CURRENT RESEARCH

The current research aimed to explore the relationship between societal freedom and conspiracy theory endorsement by examining both objective measures and individuals' subjective perceptions of societal freedom. At the macro level, we used the Human Freedom Index (HFI) as an objective measure of societal freedom, which encompasses several dimensions, including personal, civil and economic freedom. At the individual level, we focused on subjective perceptions of societal freedom. By examining these relationships, we aimed to develop a more nuanced understanding of how societal conditions and individual experiences of freedom may be related to the adoption of alternative explanatory frameworks, such as conspiracy theories.

Our research was structured through five different studies, each designed to shed light on different aspects of how societal freedom is related to conspiracy beliefs. Study 1 examined the negative relationship between objective societal freedom (as measured by the HFI) and conspiracy beliefs using a large cross‐national dataset that included participants from 36 countries (Hornsey, Pearson, et al., 2023). Study 2 assessed the robustness of these findings using a second cross‐national dataset collected by van Bavel et al. (2022). Our analyses focused on interest groups‐related COVID‐19 conspiracy beliefs, which, unlike the measure used in Study 1, do not explicitly refer to government actors.

Study 3 shifted the focus to the individual level within a single country (Poland) and examined whether individuals' perceptions of societal freedom predicted beliefs in a variety of conspiracy theories. Extending these findings, Study 4 used an experimental design to test the causal relationship between perceived societal freedom and conspiracy beliefs. Participants were randomly assigned to conditions emphasizing either high or low societal freedom (or a control condition). Finally, in Study 5, we cross‐sectionally tested the psychological mechanisms (political anger and feeling of lack of control) that mediate the relationship between perceived societal freedom and conspiracy beliefs, thereby laying the groundwork for future studies in this area. All analyses described here were conducted in R Studio (Posit Team, 2025), R version 4.5.1 (R Core Team, 2025). Data and R scripts for all studies are available on the OSF: https://osf.io/dkczs/.

## STUDY 1

The primary objective of Study 1 was to examine the relationship between generic conspiracy beliefs and the HFI, which is an objective composite measure of societal freedom. Specifically, we hypothesized that higher levels of HFI would be associated with lower levels of conspiracy beliefs.

### Method

#### Participants

From the initial sample collected by Hornsey, Pearson, et al. (2023), *N* = 6723, we excluded participants with missing data. This resulted in the final sample of *N* = 6353 individuals (65.6% women, 34.4% men, M_age_ = 21.81, SD_age_ = 5.62) from 36 countries across six continents. Detailed information on demographics and the entire study is available on the project's OSF (https://osf.io/btmnv/).

#### Measures

*Generic conspiracy beliefs* were assessed using a validated single‐item measure (Lantian et al., 2016). Participants were presented with a description stating that “Some political and social events are debated. It is suggested that the “official version” of these events could be an attempt to hide the truth from the public. This “official version” could mask the fact that these events have been planned and secretly prepared by a covert alliance of powerful individuals or organizations (for example secret services or government). What do you think?”. Then they were asked to rate the extent to which they agreed with the statement: “I think that the official version of the events given by the authorities very often hides the truth” on a scale ranging from 1 (Completely false) to 9 (Completely true).

*The Human Freedom Index (HFI)* provides a broad measure of freedom, defined as the absence of coercive constraints and is composed of 76 indicators in 12 key areas: rule of law, security and safety, movement, religion, association, assembly, and civil society, expression and information, identity and relationships, size of government, legal system and property rights, access to sound money, freedom to trade internationally and regulation of credit, labour, and business. Each indicator contributes to a comprehensive assessment of a country's freedom. Scores on the HFI range from 0 to 10, with higher scores indicating greater levels of freedom. The final score for each country is an average of the scores across all indicators. For this study, we used the publicly available HFI scores from the 2019 report (Vásquez & Porčnik, 2019), which was published closest to the data collection period.^1^

*Covariates*: In our analyses the following demographic variables were included as covariates: age and binary coded gender.

#### Analytic strategy

Given the hierarchical structure of our data, with individuals nested within countries, we applied a multilevel modelling approach. Mixed‐effects linear regression analyses were conducted using the lmerTest package (Kuznetsova et al., 2017) in R Studio, including a random intercept for countries. Continuous predictors were centred on the grand mean. *P*‐values were calculated using degrees of freedom obtained by a Satterthwaite approximation, and standardized coefficients were computed using the sjPlot package (Lüdecke, 2023). Our analysis consisted of three steps. First, we estimated Model 0, a baseline model that included only the random intercept. In Model 1, we included the individual‐level predictors. Finally, in Model 2, we added our key country‐level predictor, the HFI, to assess its effect on generic conspiracy beliefs.

### Results

**FIGURE 1:**
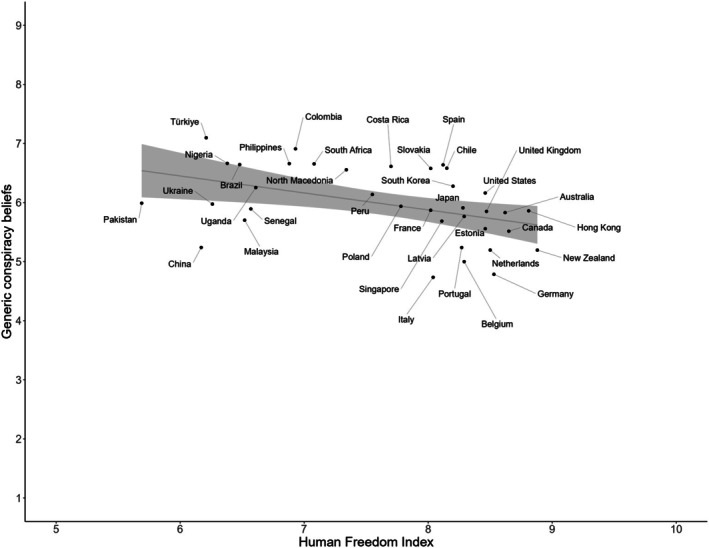
Country‐level relationship between generic conspiracy beliefs and the Human Freedom Index. To improve clarity, the x‐axis has been trimmed to show values between 5 and 10.

Table S1 presents descriptive statistics and correlations among all individual‐level variables included in this study. We began by plotting the direct relationship between country‐level means of generic conspiracy beliefs and the HFI. As shown in Figure 1, the observed effect was negative.

To formally test our hypothesis, in the next step, we tested the series of multilevel models (Table 1). In Model 1, we included individual‐level predictors and observed a significant improvement in model fit compared to Model 0, χ^2^ (2) = 21.75, *p* < .001. Gender was a positive predictor of generic conspiracy beliefs (*β* = .06), while the effect of age was not significant (*β* = −.01). In the second model, we introduced our key predictor, the HFI, which led to a significant improvement in model fit compared to Model 1, χ^2^ (1) = 7.83, *p* = .005. Consistent with our hypothesis, the HFI emerged as a significant negative predictor of generic conspiracy beliefs (*β* = −.14). Across the models, the individual‐level effects remained stable.

**TABLE 1:** Individual‐level and country‐level predictors of generic conspiracy beliefs.

Predictors	Model 0		Model 1		Model 2	
	*B* (SE)	*p*	*B* (SE)	*p*	*B* (SE)	*p*
(intercept)	5.98 (0.10)	<.001	5.80 (0.11)	<.001	5.80 (0.10)	<.001
Individual‐level effects						
Age			−0.004 (0.01)	.454	−0.004 (0.01)	.485
Gender			0.25 (0.06)	<.001	0.26 (0.06)	<.001
Country‐level effects						
Human Freedom Index (HFI)					−0.31 (0.11)	.007
Random effects						
σ^2^	3.80		3.78		3.78	
*τ*_*00*_	0.35 _Country_		0.37 _Country_		0.30 _Country_	
ICC	.08		.09		.07	
*N* (countries)	36		36		36	
Observations	6353		6353		6353	
Marginal *R* ^2^	.00		.004		.02	
Conditional *R* ^2^	.08		.09		.09	
Deviance	26,599		26,577		26,570	

*Note*: Gender was a dichotomous variable with values of 0 = man and 1 = woman.

### Discussion

Our results supported the main hypothesis by showing a significant negative relationship between the HFI and generic conspiracy beliefs. However, the main limitation of this study is the use of the Lantian et al. (2016) conspiracy belief measure, which explicitly refers to government actors. While research suggests that conspiracy beliefs may reflect responses to environmental cues about institutional misconduct (Alper, 2023; Alper & Imhoff, 2023), this measure's mention of government authorities makes it difficult to determine whether our findings can be generalized to broader patterns of conspiratorial thinking or are specific to government‐related suspicions. This concern motivated Study 2, which examined COVID‐19‐related conspiracy beliefs.

## STUDY 2

Study 2 sought to build on the findings of Study 1 by examining a different type of conspiracy content: interest groups‐related COVID‐19 conspiracy beliefs. Unlike the conspiracy belief measure used in Study 1, which was explicitly related to government actors, this item from the COVID‐19 conspiracy measure assessed conspiracy beliefs that are not necessarily associated with government distrust. This allowed us to test whether the observed relationship between societal freedom and conspiracy beliefs extends to broader conspiracy content beyond those specifically targeting government actors.

### Method

#### Participants

We used data from an international collaborative project conducted during the early stages of the COVID‐19 pandemic (van Bavel et al., 2022). From an initial sample of countries, those with at least 100 participants who completed the questionnaire were included in the study. After excluding respondents with missing data on key variables and Cuba due to a lack of available HFI data, the final sample included 44,458 individuals (51.9% women, 48.1% men; M_age_ = 43.23, SD_age_ = 16.10) from 52 countries. Detailed information on ethical approval, informed consent, and methodological procedures is available in the project's Open Science Framework repository (https://osf.io/y7ckt/).

#### Measures

*Interest groups‐related COVID‐19 conspiracy beliefs* were measured using one item from a scale prepared by van Bavel et al. (2022). It assessed the extent to which participants believed that “The coronavirus (COVID‐19) is a hoax invented by interest groups for financial gains”. Responses were provided on an 11‐point scale ranging from 0 (strongly disagree) to 10 (strongly agree).

*The Human Freedom Index (HFI)* scores were prepared by the same organization as in Study 1. This time, however, we sourced them from the report published in 2020 (Vásquez & McMahon, 2020), as it was closest to the data collection period.

*Covariates*: In the analyses we controlled for age, gender and political ideology. Gender was coded as a binary variable with values coded as 0 (man) and 1 (woman). Political ideology was assessed using a 11‐point scale ranging from 0 (extremely liberal/left‐leaning) to 10 (extremely conservative/right‐leaning) and we included it as prior research suggests that ideological orientation and extremity may affect susceptibility to conspiracy beliefs (Imhoff et al., 2022; Van Prooijen et al., 2015).^2^

#### Analytic strategy

As in Study 1, we used multilevel modelling to account for the hierarchical structure of the data, with individuals nested within countries. Mixed‐effects linear regression analyses were conducted using the lmerTest package (Kuznetsova et al., 2017) in R Studio, with a random intercept specified for countries. Continuous predictors were centred on the grand mean. *P*‐values were calculated using degrees of freedom estimated using the Satterthwaite approximation and standardized coefficients were computed using the sjPlot package (Lüdecke, 2023). First, we estimated a null model (Model 0) containing only the random intercept to assess the extent of between‐country variability in conspiracy beliefs. Next, in Model 1, we added individual‐level predictors. Finally, in Model 2, we introduced the key country‐level predictor, the HFI to examine its association with COVID‐19 conspiracy beliefs while controlling for individual‐level covariates.

### Results

**FIGURE 2:**
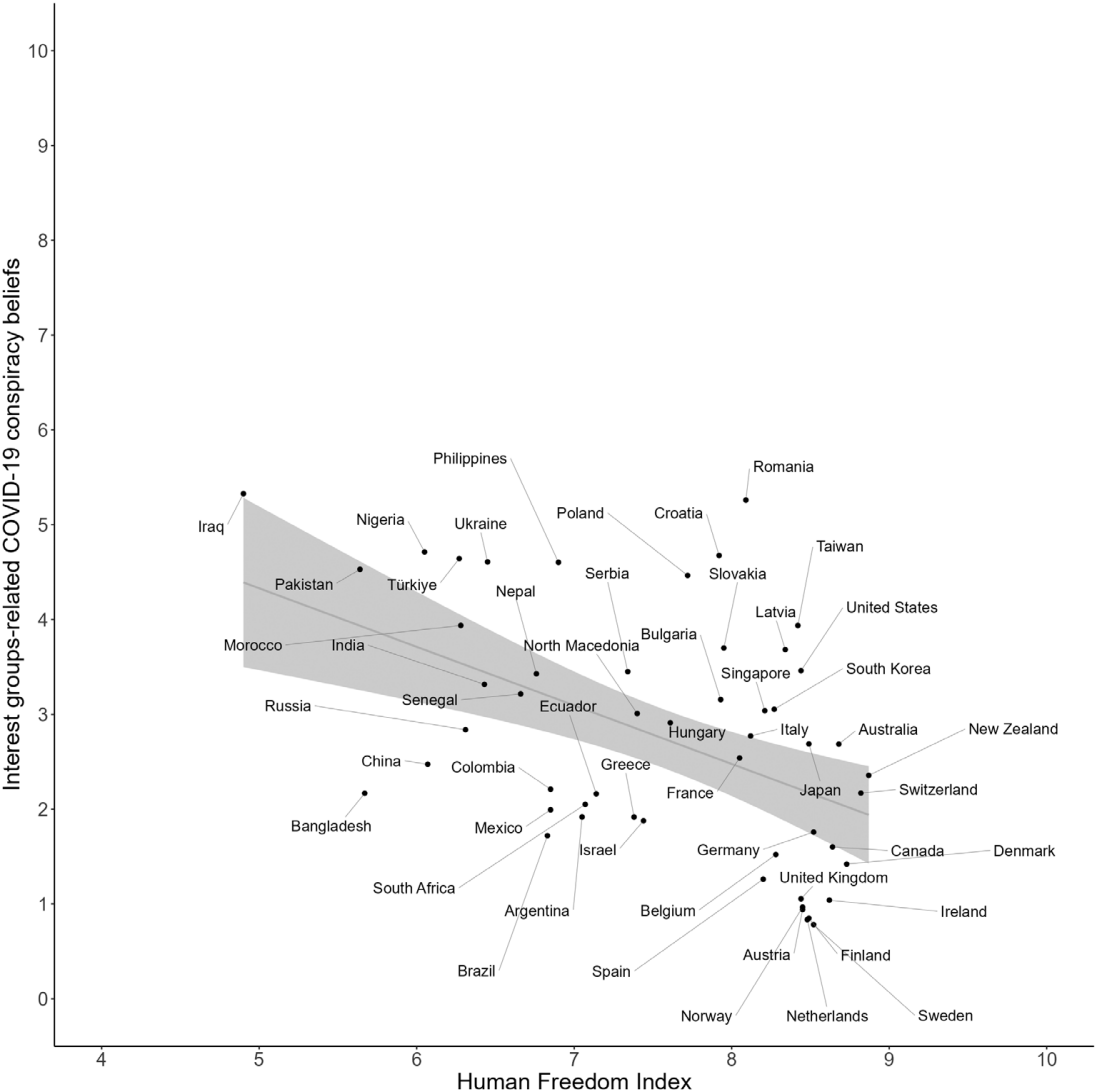
Country‐level relationship between interest groups‐related COVID‐19 conspiracy beliefs and the Human Freedom Index. To improve clarity, the x‐axis has been trimmed to show values between 4 and 10.

Descriptive statistics and correlations between the variables studied are presented in Table S3. To examine the relationship between societal freedom and COVID‐19 interest groups‐related conspiracy beliefs, we began with a preliminary analysis of their country‐level means. Figure 2 illustrates the negative relationship between HFI scores and the prevalence of these conspiracy beliefs across countries included in our sample.

Our next step was the testing of a series of multilevel models (Table 2). Model 1 included individual‐level predictors (age, gender and political ideology) and significantly improved model fit compared to Model 0, χ^2^ (3) = 1210.82, *p* < .001. Age emerged as a negative predictor of conspiracy beliefs (*β* = −.04), while the effect of gender was not significant (*β* = .004). In addition, the effect of political orientation was also significant (*β* = .15), with higher scores on this scale associated with higher interest groups‐related COVID‐19 conspiracy beliefs. In Model 2, the HFI was introduced as a country‐level predictor, resulting in a significant improvement in model fit compared to Model 1, χ^2^ (1) = 13.90, *p* < .001. As hypothesized, the HFI was a significant negative predictor of interest groups‐related COVID‐19 conspiracy beliefs (*β* = −.17) The effects of age, gender and political orientation remained consistent across models.

**TABLE 2:** Individual and country‐level predictors of interest groups‐related COVID‐19 conspiracy beliefs.

Predictors	Model 0		Model 1		Model 2	
	*B* (SE)	*p*	*B* (SE)	*p*	*B* (SE)	*p*
(intercept)	2.74 (0.17)	<.001	2.72 (0.16)	<.001	2.63 (0.15)	<.001
Individual‐level effects						
Age			−0.01 (0.001)	<.001	−0.01 (0.001)	<.001
Gender			0.03 (0.03)	.323	0.03 (0.03)	.320
Political orientation			0.21 (0.01)	<.001	0.21 (0.01)	<.001
Country‐level effects						
Human Freedom Index (HFI)					−0.58 (0.15)	<.001
Random effects						
*σ* ^2^	8.52		8.29		8.29	
*τ*_*00*_	1.58 _Country_		1.39 _Country_		1.08 _Country_	
ICC	.16		.14		.12	
*N* (countries)	52		52		52	
Observations	44,458		44,458		44,458	
Marginal *R* ^*2*^	.00		.03		.06	
Conditional *R* ^*2*^	.16		.17		.17	
Deviance	221,649		220,438		220,424	

*Note*: Gender was a dichotomous variable with values of 0 = man and 1 = woman.

To increase the robustness of our findings, we also conducted analyses using the full COVID‐19 conspiracy belief scale (see the Data S1). These analyses yielded results consistent with our main findings: the HFI had a significant negative effect on COVID‐19 conspiracy beliefs (*β* = −.16, *p* = .002).

### Discussion

Study 2 extended the findings of Study 1 by using a measure of conspiracy beliefs that did not explicitly refer to the government, enabling us to examine whether the relationship between societal freedom and conspiracy beliefs extends to conspiratorial thinking that is not government‐specific. Consistent with our hypothesis, the results showed that individuals living in countries with greater societal freedom were less likely to endorse COVID‐19 conspiracy beliefs, even when these beliefs targeted interest groups rather than governmental institutions.

## STUDY 3

In Study 3, we aimed to extend previous cross‐national research by examining the relationship between perceived societal freedom at the individual level and various conspiracy beliefs. By examining this relationship in the Polish context, we seek to understand how individuals' perceptions of societal freedom are related to belief in conspiracy theories. We hypothesized that perceived societal freedom would be negatively associated with generic conspiracy beliefs (H1a), conspiracy mentality (H1b), generic conspiracist beliefs (H1c), conspiracy beliefs about financial crises (H1d), as well as vaccine‐related conspiracy beliefs (H1e), after controlling for age, gender and political orientation. This study has been preregistered with the details available on the OSF (https://osf.io/nupek/).

### Method

#### Participants

Participants were recruited through an online panel administered by an external company (Pollster). We targeted a sample of 264 participants to detect an increase in model *R* ^2^ with an effect size of *f* ^2^ = 0.03, an alpha level of .05 and a power of .80 (Faul et al., 2007). To ensure data quality, we implemented several exclusion criteria. Participants were excluded if they did not respond to at least 80% of the items in each scale or did not respond to at least one of the two attention checks. After applying these exclusion criteria, the final sample consisted of 278 participants (38.5% women, 61.5% men, M_age_ = 43.63, SD_age_ = 15.84). Ethical approval for this study was obtained from the Research Ethics Committee of the Jagiellonian University (221.6120.39.2024).

#### Measures

*Perceived societal freedom* was measured using an 8‐item scale developed based on indicators from the HFI (*α* = .88). The scale assessed participants' perceptions of various domains of freedom in their country, including rule of law, security and safety, movement, religion, association and assembly, expression and information, relationships and economic freedom. Participants rated each item on a scale from 1 (not at all) to 7 (very much so), with higher scores indicating greater perceived societal freedom. Detailed descriptions of each item can be found in the Data S1.

*Generic conspiracy beliefs* were assessed using the same single‐item measure as in Study 1 (Lantian et al., 2016). This time, the scale ranged from 1 (Completely false) to 7 (Completely true).

*Conspiracy mentality* was measured using the Polish adaptation of Imhoff and Bruder's (2014) Conspiracy Mentality Scale. The scale consisted of five items (*α* = .90), and participants responded on a scale ranging from 0 (complete disagreement) to 100 (complete agreement).

*Vaccine‐related conspiracy beliefs* were assessed with a single‐item measure (Toribio‐Flórez et al., 2025) that was included in the Trust in Science Project (Mede et al., 2025). Participants rated their agreement with the statement, “Scientists work together to cover up the dangers of vaccines” on a 7‐point Likert scale ranging from 1 (strongly disagree) to 7 (strongly agree).

*Generic conspiracist beliefs* were measured using a 5‐item scale developed by Kay and Slovic (2023). Participants indicated their agreement with each statement on a 6‐point Likert scale ranging from 1 (strongly disagree) to 6 (strongly agree). An example item from the scale reads: “Experiments involving new drugs or technologies are routinely carried out on the public without their knowledge or consent” (*α* = .88).

*Conspiracy beliefs about financial crises* were measured using an adapted version of van Prooijen et al.'s (2015) financial conspiracy beliefs scale. To capture beliefs about financial crises in general, the original scale was modified to reflect more general statements about the causes, consequences and origins of financial crises. Participants read the following instructions: “From time to time, countries around the world experience financial crises that have far‐reaching effects on the economic lives of citizens and on the infrastructure of the countries affected by such financial downturns. Please read the following statements about the origins, causes, or consequences of financial crises and rate your agreement using a scale from 1 (strongly disagree) to 7 (strongly agree).” and answered six items (*α* = .94), e.g., “Financial crises have been caused by some banks to win the competition from other banks” (see the Data S1 for a complete list of all items).

*Covariates*: In all analyses we controlled for participants' age, binary‐coded gender: 0 (woman), 1 (man) and political orientation, which was measured on a 7‐point scale ranging from 1 (very left‐wing) to 7 (very right‐wing).

### Results

Means, standard deviations and correlations of all variables included in the study are presented in Table S7. To verify our hypotheses, we tested a series of hierarchical linear regression models predicting different types of conspiracy beliefs.

#### Generic conspiracy beliefs

In Model 1 (Table 3), we included all covariates. Political orientation was a significant positive predictor of generic conspiracy beliefs (*β* = .18), while the effects of gender (*β* = −.05) and age (*β* = −.02) were not significant. In Model 2, we added perceived societal freedom and found that it was negatively associated with generic conspiracy beliefs (*β* = −.07). However, this effect did not reach significance and did not support our hypothesis (H1a). The effects of the covariates were consistent with those in Model 1.

**TABLE 3:** Hierarchical regression results– Generic conspiracy beliefs as the DV.

	Model 1			95% CI		Model 2			95% CI	
	*B*	SE	*p*	LL	UL	*B*	SE	*p*	LL	UL
(intercept)	4.34	0.40	<.001	3.56	5.12	4.84	0.57	<.001	3.71	5.96
Gender	−0.18	0.20	.384	−0.58	0.22	−0.17	0.20	.397	−0.58	0.23
Age	−0.002	0.01	.746	−0.01	0.01	−0.001	0.01	.880	−0.01	0.01
Political orientation	0.19	0.06	.002	0.07	0.31	0.18	0.06	.003	0.06	0.31
Perceived societal freedom						−0.10	0.09	.233	−0.27	0.07
*R* ^*2*^	.036					.041				
*F*	3.45		.017			2.95		.021		
Δ*R* ^*2*^						.005				
Δ*F*						1.43		.233		

#### Conspiracy mentality

In Model 1 (Table 4), political orientation was found to be a strong positive predictor of conspiracy mentality (*β* = .33). Furthermore, gender was also found to have a significant effect, with men reporting lower levels of conspiracy mentality than women (*β* = −.17). The effect of age was not significant (*β* = .001). In Model 2, when perceived societal freedom was added, it had a significant negative effect on conspiracy mentality (*β* = −.13), supporting our hypothesis (H1b). The effects of the covariates remained stable across models.

**TABLE 4:** Hierarchical regression results– Conspiracy mentality as the DV.

	Model 1			95% CI		Model 2			95% CI	
	*B*	SE	*p*	LL	UL	*B*	SE	*p*	LL	UL
(intercept)	52.92	5.14	<.001	42.80	63.05	65.61	7.39	<.001	51.07	80.16
Gender	−8.01	2.66	.003	−13.25	−2.77	−7.88	2.64	.003	−13.08	−2.68
Age	0.001	0.08	.988	−0.16	0.16	0.03	0.08	.724	−0.13	0.19
Political orientation	4.74	0.81	<.001	3.15	6.33	4.56	0.80	<.001	2.97	6.14
Perceived societal freedom						−2.63	1.11	.018	−4.81	−0.45
*R* ^2^	.13					.15				
*F*	14		<.001			12.08		<.001		
Δ*R* ^2^						.02				
Δ*F*						5.63		.018		

#### Generic conspiracist beliefs

Model 1 (Table 5), which included all the covariates, revealed that political orientation was a positive predictor of generic conspiracist beliefs (*β* = .23). Neither gender (*β* = −.04) nor age (*β* = −.07) had a significant effect. Model 2, which included perceived societal freedom, showed that, in line with our hypothesis (H1c), this variable had a significant negative effect on generic conspiracist beliefs (*β* = −.14). There were no notable changes to the effects of the covariates.

**TABLE 5:** Hierarchical regression results ‐ Generic conspiracist beliefs as the DV.

	Model 1			95% CI		Model 2			95% CI	
	*B*	SE	*p*	LL	UL	*B*	SE	*p*	LL	UL
(intercept)	2.80	0.33	<.001	2.15	3.45	3.63	0.47	<.001	2.70	4.57
Gender	−0.12	0.17	.489	−0.46	0.22	−0.11	0.17	.518	−0.44	0.22
Age	−0.01	0.01	.254	−0.02	0.004	−0.004	0.01	.427	−0.01	0.01
Political orientation	0.20	0.05	<.001	0.10	0.30	0.19	0.05	<.001	0.09	0.29
Perceived societal freedom						−0.17	0.07	.016	−0.31	−0.03
*R* ^2^	.06					.08				
*F*	5.77		<.001			5.88		<.001		
Δ*R* ^2^						.02				
Δ*F*						5.91		.016		

#### Vaccine‐related conspiracy beliefs

In Model 1 (Table 6), political orientation was a strong positive predictor of vaccine‐related conspiracy beliefs (*β* = .29), while the effects of gender (*β* = −.07) and age (*β* = −.01) were not significant. In Model 2, we included perceived societal freedom, which in line with H1e was significantly associated with lower levels of vaccine‐related conspiracy beliefs (*β* = −.18). The effects of the other variables remained largely unchanged.

**TABLE 6:** Hierarchical regression results– Vaccine‐related conspiracy beliefs as the DV.

	Model 1			95% CI		Model 2			95% CI	
	*B*	SE	*p*	LL	UL	*B*	SE	*p*	LL	UL
(intercept)	2.79	0.49	<.001	1.84	3.75	4.38	0.69	<.001	3.02	5.75
Gender	−0.28	0.25	.266	−0.77	0.21	−0.26	0.25	.286	−0.75	0.22
Age	−0.002	0.01	.824	−0.02	0.01	0.002	0.01	.819	−0.01	0.02
Political orientation	0.38	0.08	<.001	0.23	0.53	0.35	0.08	<.001	0.21	0.50
Perceived societal freedom						−0.33	0.10	.002	−0.53	−0.13
*R* ^2^	.09					.12				
*F*	8.48		<.001			9.11		<.001		
Δ*R* ^2^						.03				
Δ*F*						10.14		.002		

#### Conspiracy beliefs about financial crises

The results of Model 1 (Table 7) showed that political orientation was a significant positive predictor of conspiracy beliefs about financial crises (*β* = .29). However, the effects of gender (*β* = −.07) and age (*β* = .02) were not significant. In Model 2, when perceived societal freedom was added, it showed a significant negative association with conspiracy beliefs about financial crises (*β* = −.14), supporting our hypothesis (H1d). The addition of this variable did not substantially alter the effects of the other covariates.

**TABLE 7:** Hierarchical regression results– Conspiracy beliefs about financial crises as the DV.

	Model 1			95% CI		Model 2			95% CI	
	*B*	SE	*p*	LL	UL	*B*	SE	*p*	LL	UL
(intercept)	3.26	0.38	<.001	2.52	4.01	4.22	0.55	<.001	3.15	5.30
Gender	−0.22	0.20	.270	−0.60	0.17	−0.21	0.19	.288	−0.59	0.18
Age	0.002	0.01	.790	−0.01	0.01	0.004	0.01	.540	−0.01	0.02
Political orientation	0.30	0.06	<.001	0.18	0.41	0.28	0.06	<.001	0.17	0.40
Perceived societal freedom						−0.20	0.08	.015	−0.36	−0.04
*R* ^2^	.09					.11				
*F*	8.52		<.001			7.99		<.001		
Δ*R* ^2^						.02				
Δ*F*						5.94		.015		

### Discussion

The present study extended previous cross‐national findings by examining the relationship between perceived societal freedom and conspiracy beliefs at the individual level. Our results showed that individuals' perceptions of freedom in their society are associated with lower endorsement of various conspiracy beliefs. This relationship was consistently observed across multiple domains, including conspiracy mentality, vaccine‐related conspiracy beliefs and conspiracy beliefs about financial crises. Although the effect for generic conspiracy beliefs did not reach statistical significance, it was negative in line with our predictions. However, a key limitation of this study is the inability to determine causality due to its correlational design, leaving the direction of the observed relationships unresolved, which motivated us to conduct Study 4.

## STUDY 4

The correlational nature of the previous study precluded causal inferences about the relationship between the studied variables. Study 4 addressed this limitation by experimentally manipulating perceived societal freedom to test its causal effect on conspiracy beliefs. Based on our previous findings, we hypothesized that participants in the low‐freedom condition would report higher levels of generic conspiracist beliefs (H1a), conspiracy beliefs about financial crises (H1b) and vaccine‐related conspiracy beliefs (H1c) than participants in the high‐freedom and control conditions. Conversely, we predicted that participants in the high‐freedom condition would report significantly lower levels of conspiracy beliefs in all measured domains: generic (H2a), financial (H2b) and vaccine‐related (H2c). In all analyses we included the same covariates as in Study 3 (age, binary‐coded gender and political orientation). This study has been preregistered on the Open Science Framework (https://osf.io/89wrt/overview?view_only=43fe7bb40fe149aa89e95cbb41cb554e).

### Method

#### Participants

Once again participants were recruited through an online panel. A priori power analysis using G*Power (Faul et al., 2007) indicated that a sample size of 244 participants would provide sufficient statistical power (.80) to detect an effect size of *f* = 0.2 with an alpha level of .05 and three covariates included in the analyses. To account for potential exclusions due to missing data or failed attention checks, we collected data from 280 individuals. Although our preregistration specified data collection from 260 individuals, we increased the sample size because only 230 of the responses from the sample indicated by the preregistered stopping rule were valid after applying the exclusion criteria and additional data collection was necessary to reach our target sample size. Participants were excluded if they failed to respond to at least 80% of the items in each scale or failed at least one of the two attention checks included in the survey. After applying these preregistered exclusion criteria, the final sample consisted of 246 participants (52.8% women, 47.2% men, M_age_ = 41.66, SD_age_ = 17.15). 77 respondents were assigned to the high‐freedom condition, 94 to the control group and 75 to the low‐freedom condition. Ethical approval for this study was obtained from the Research Ethics Committee of the Jagiellonian University (221.6120.39.2024).

#### Procedure and design

After providing informed consent, participants were randomly assigned to one of three experimental conditions: low freedom, high freedom, or control. In the low‐freedom condition, participants were asked to think about and describe examples of mandatory rules and policies in Poland that limit individual choice. The instructions emphasized situations in which people have no choice but to comply with rules, regardless of their personal preferences. Participants were asked to write three examples from their current lives in which they had to comply with such regulations, had no choice and felt more restricted than citizens of other countries in the same situation.

In the high‐freedom condition, participants were asked to generate examples of voluntary regulations and policies in Poland that provide individuals with multiple choices. The instructions emphasized situations in which people have multiple options for complying with rules. Participants were asked to provide three examples from their current lives in which they could choose from several solutions recommended by such regulations and felt less restricted compared to citizens of other countries in the same situation.

Participants in both experimental conditions were given fixed time to complete the task and could not move on to the next section until the three minutes were up. In the control condition, they proceeded directly to the dependent variables. Because we expected that the effect of our manipulation might diminish with the amount of time spent answering the conspiracy‐related questions, we selected for this study three of the conspiracy belief measures included in Study 3 that we found to be the most diverse in terms of topics covered.

#### Measures

Generic conspiracist beliefs (*α* = .79), vaccine‐related conspiracy beliefs and conspiracy beliefs about financial crises (*α* = .94) were assessed using the same measures as in Study 3. In each analysis we controlled for age, binary‐coded gender (0 = woman, 1 = man) and political orientation measured on a scale ranging from 1 (very left‐wing) to 7 (very right‐wing).

### Results

We conducted a series of one‐way ANCOVAs to examine the effect of the manipulation of perceived societal freedom on the three types of conspiracy beliefs: generic conspiracist beliefs, conspiracy beliefs about financial crises, and vaccine‐related conspiracy beliefs. Table S13 provides an overview of the descriptive statistics and correlations between the variables included in this study.

#### Generic conspiracist beliefs

**FIGURE 3:**
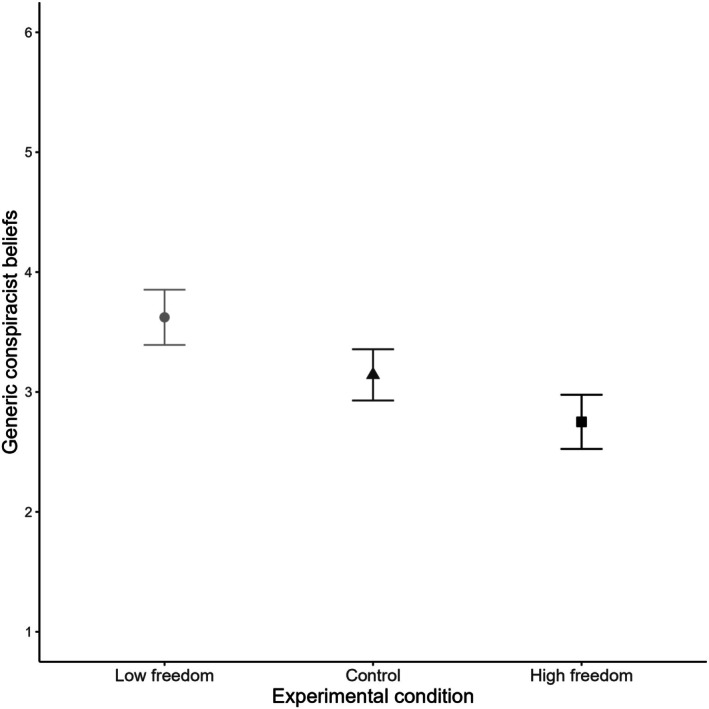
Experimental condition and average scores of generic conspiracist beliefs. Error bars represent 95% Confidence Intervals.

For generic conspiracist beliefs, the ANCOVA revealed a significant main effect of condition, *F*(2, 240) = 14.54, *p* < .001, *η*_p_^2^ = .11. Tukey's post‐hoc comparisons provided support for our hypotheses, as consistent with H1a, participants in the low‐freedom condition reported significantly higher generic conspiracist beliefs (M = 3.62, SE = 0.12) compared to both the control condition (M = 3.14, SE = 0.11, *p* = .011, *d* = 0.49) and the high‐freedom condition (M = 2.75, SE = 0.12, *p* < .001, *d* = 0.89). In addition, the difference between the control and high‐freedom conditions was also significant (*p* = .045, *d* = 0.40), supporting Hypothesis H2a. The summarized results are presented in Figure 3, with additional details included in Table S14.

#### Conspiracy beliefs about financial crises

**FIGURE 4:**
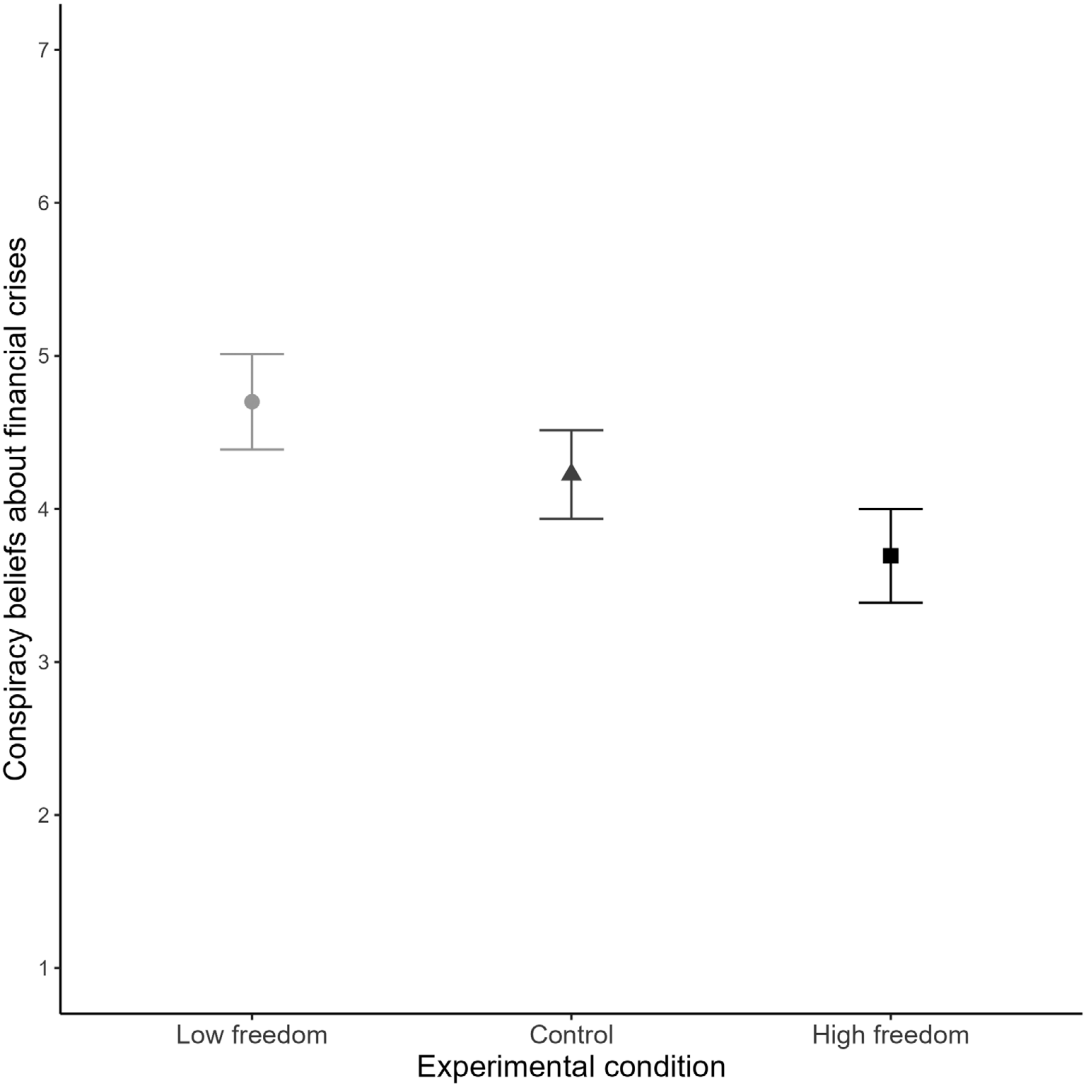
Experimental condition and average scores of conspiracy beliefs about financial crises. Error bars represent 95% Confidence Intervals.

In the case of conspiracy beliefs about financial crises, a similar ANCOVA indicated a significant main effect of condition, *F*(2, 240) = 10.60, *p* < .001, *η*_p_^2^ = .08. Tukey's post‐hoc tests indicated that participants in the high‐freedom condition reported significantly lower levels of conspiracy beliefs about financial crises (M = 3.69, SE = 0.16) compared to those in the low‐freedom (M = 4.70, SE = 0.16, *p* < .001, *d* = 0.76) and control (M = 4.22, SE = 0.15, *p* = .044, *d* = 0.40) conditions, supporting H2b. However, contrary to hypothesis H1b, the difference between the low‐freedom and control conditions was not significant (*p* = .085, *d* = 0.36). The summarized results are shown in Figure 4, with further details provided in Table S15.

#### Vaccine‐related conspiracy beliefs

**FIGURE 5:**
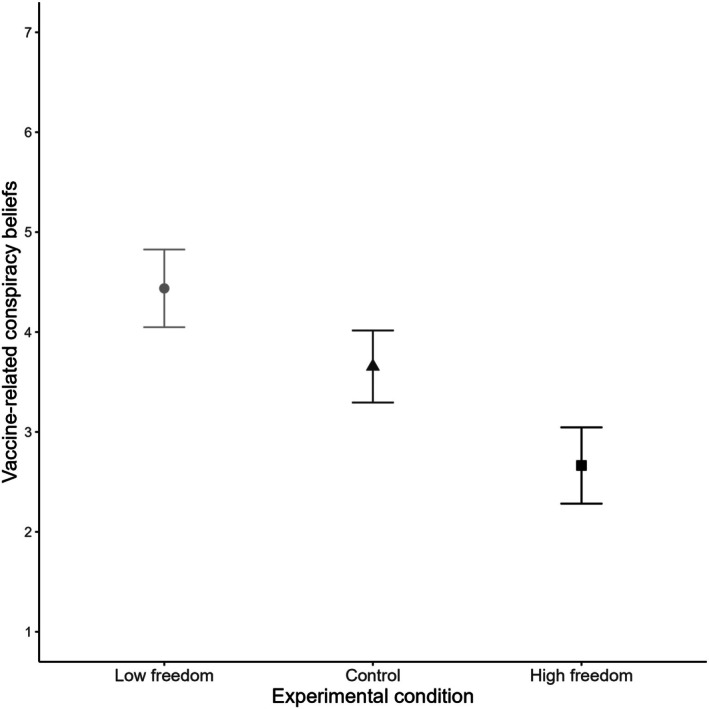
Experimental condition and average scores of vaccine‐related conspiracy beliefs. Error bars represent 95% Confidence Intervals.

The ANCOVA for vaccine‐related conspiracy beliefs revealed a significant main effect of the experimental condition, *F*(2, 240) = 21.18, *p* < .001, *η*_p_^2^ = .15. Tukey's post‐hoc comparisons indicated that participants in the low‐freedom condition reported significantly higher vaccine‐related conspiracy beliefs (M = 4.44, SE = 0.20) compared to both the control condition (M = 3.65, SE = 0.18, *p* = .015, *d* = 0.47) and the high‐freedom condition (M = 2.66, SE = 0.19, *p* < .001, *d* = 1.07). The difference between the control and high‐freedom conditions was also statistically significant (*p* = .001, *d* = 0.60), fully supporting hypotheses H1c and H2c. Figure 5 presents the summarized results, with additional information included in Table S16.

### Discussion

In this experimental study, we provided evidence for causality in the relationship between perceived social freedom and conspiracy beliefs. The results supported our hypotheses by showing that participants in the high‐freedom condition reported significantly lower levels of generic, financial and vaccine‐related conspiracy beliefs compared to the low‐freedom and control conditions. Although the difference between the low‐freedom and control conditions for conspiracy beliefs about financial crises did not reach significance, the overall results provide compelling evidence for the notion that a perceived lack of societal constraints translates into lower levels of conspiratorial thinking.

## STUDY 5

Study 4 provided evidence of the causal effect of perceived societal freedom on conspiracy beliefs. However, it did not examine the psychological mechanisms underlying this effect. To address this, Study 5 employed a cross‐sectional correlational design to gather preliminary evidence on the potential psychological mechanisms underlying our main findings. Based on our theoretical framework, we identified two potential mediators: political anger and feelings of a lack of control. We hypothesized that greater perceived societal freedom would be associated with lower levels of political anger (H1a) and reduced feelings of a lack of control (H1b), both of which would, in turn, be associated with a higher level of endorsement of four types of conspiracy theories (H2a‐H2d).

### Method

#### Participants

Once again, participants were recruited through an online panel managed by an external company (Pollster). The target sample size of 590 individuals was determined using Schoemann et al.'s (2017) tool, with the aim of detecting indirect effects with a power of .80 at an alpha level of .05. The expected effect size and standard deviations for some variables were estimated based on a previous correlational study (Study 3); for the remaining variables, values of standard deviation were set to 1.5. To ensure data quality, we excluded participants if they did not respond correctly to at least one of three attention checks. After applying this exclusion criterion, the final sample consisted of 592 participants (52.7% women, 47.3% men, M_age_ = 47.74, SD_age_ = 16.40). Ethical approval for this study was obtained from the Research Ethics Committee of the Jagiellonian University (221.6120.39.2024).

#### Measures

Perceived societal freedom (*α* = .88), generic conspiracy beliefs, generic conspiracist beliefs (*α* = .85), vaccine‐related conspiracy beliefs, conspiracy beliefs about financial crises (*α* = .96) and covariates (age, binary‐coded gender, political orientation) were assessed using the same measures as in previous studies.

*Lack of control* was assessed using the 7‐item Sense of Mastery Scale (Pearlin & Schooler, 1978; *α* = 81). Participants responded to statements such as “I have little control over the things that happen to me” using a 7‐point Likert scale ranging from 1 (strongly disagree) to 7 (strongly agree).

*Political anger* was measured using items adapted from Lambert et al. (2019). Participants were instructed to think about the current political situation in Poland and rate the extent to which they felt angry, mad, irate and furious (*α* = .94) on a 7‐point scale ranging from 1 (not at all) to 7 (very much).

#### Analytic strategy

To test our hypotheses, we specified four mediation models, each of which tested the same two mediators— lack of control and political anger— as pathways linking perceived societal freedom to different conspiracy belief outcomes: generic conspiracy beliefs, generic conspiracist beliefs, vaccine‐related conspiracy beliefs and conspiracy beliefs about financial crises. Each model included age, gender and political orientation as covariates. To calculate them we used the PROCESS macro for R (Hayes, 2022; Model 4) and assessed the significance of indirect effects using bootstrapped (5000 re‐samples) 95% Confidence Intervals.

### Results

Table S20 presents means, standard deviations and correlations of all the variables included in the study. Across all models, perceived societal freedom significantly predicted both mediators. It was associated with a lower sense of lack of control (*B* = −0.22, SE = 0.04, *p* < .001) and lower levels of political anger (*B* = −0.22, SE = 0.06, *p* < .001). Among the covariates, age was negatively associated with a lack of control (*B* = −0.01, SE = 0.003, *p* = .044) and positively associated with political anger (*B* = 0.02, SE = 0.004, *p* < .001). Gender had no effect on lack of control (*B* = −0.12, SE = 0.09, *p* = .196) and political anger (*B* = 0.16, SE = 0.13, *p* = .243). Political orientation was not significantly associated with lack of control (*B* = 0.01, SE = 0.03, *p* = .848) but was positively related to political anger (*B* = 0.12, SE = 0.04, *p* = .006).

#### Generic conspiracy beliefs

**FIGURE 6:**
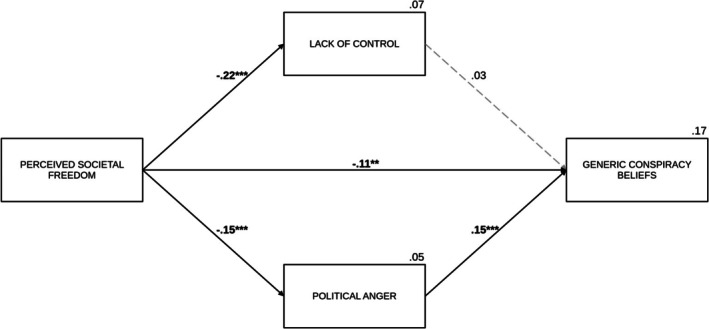
The effect of perceived societal freedom on generic conspiracy beliefs via lack of control and political anger. **p < .01. ***p < .001. The figure presents standardized coefficients. Dashed lines represent non‐significant effects. The value between the main predictor and the dependent variable indicates the direct effect. We controlled for age, gender and political orientation in the analysis.

The analysis revealed that political anger positively predicted generic conspiracy beliefs (*B* = 0.16, SE = 0.04, *p* < .001). However, the effect of lack of control was not significant (*B* = 0.04, SE = 0.06, *p* = .510). The indirect effect of societal freedom via political anger (IE = −0.03, SE_boot_ = 0.01, 95% CI [−0.07, −0.01]) was also significant, while the indirect effect through lack of control was not significant (IE = −0.01, SE_boot_ = 0.01, 95% CI [−0.03, 0.02]). After accounting for the effects of the mediators, the direct effect of perceived societal freedom on generic conspiracy beliefs was significant (*B* = −0.17, SE = 0.06, *p* = .005), similarly as the total effect (*B* = −0.21, β = −.14, SE = 0.06, *p* < .001), which indicates a partial mediation. Among the covariates, political orientation was a positive predictor (*B* = 0.36, SE = 0.04, *p* < .001) of generic conspiracy beliefs, while the effects of age (*B* = 0.001, SE = 0.004, *p* = .731) and gender (*B* = −0.15, SE = 0.13, *p* = .251) were not significant. Figure 6 presents the summary of the model with standardized coefficients.

#### Generic conspiracist beliefs

**FIGURE 7:**
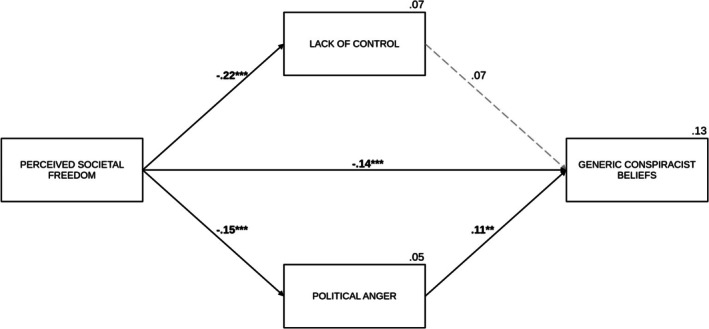
The effect of perceived societal freedom on generic conspiracist beliefs via lack of control and political anger. **p < .01. ***p < .001. The figure presents standardized coefficients. Dashed lines represent non‐significant effects. The value between the main predictor and the dependent variable indicates the direct effect. We controlled for age, gender and political orientation in the analysis.

Political anger emerged as the significant predictor of generic conspiracist beliefs (*B* = 0.09, SE = 0.03, *p* = .005), while the effect of lack of control (*B* = 0.07, SE = 0.05, *p* = .102) was not significant. The indirect effect through political anger was also significant (IE = −0.02, SE_boot_ = 0.01, 95% CI [−0.04, −0.004]), while the one via lack of control (IE = −0.02, SE_boot_ = 0.01, 95% CI [−0.04, 0.004]) was not significant. After accounting for the effects of the mediators, perceived societal freedom had a significant direct effect on generic conspiracist beliefs (*B* = −0.16, SE = 0.05, *p* < .001). The total effect was also significant (*B* = −0.20, *β* = −.17, SE = 0.05, *p* < .001). Political orientation showed a positive association (*B* = 0.22, SE = 0.03, *p* < .001) with generic conspiracist beliefs, while neither age (*B* = 0.002, SE = 0.003, *p* = .445) nor gender (*B* = −0.03, SE = 0.10, *p* = .759) was a significant predictor of this variable. See Figure 7 for a summary of the model with standardized coefficients.

#### Vaccine‐related conspiracy beliefs

**FIGURE 8:**
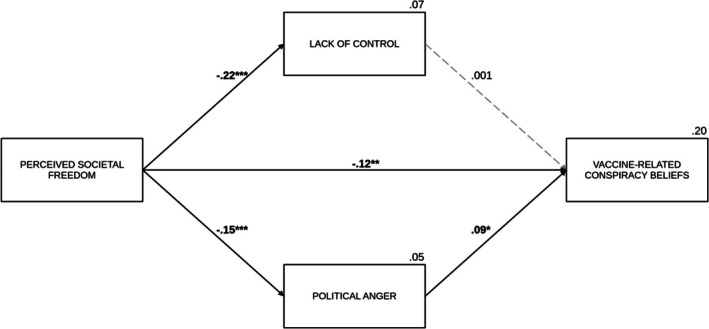
The effect of perceived societal freedom on vaccine‐related conspiracy beliefs via lack of control and political anger. *p < .05. **p < .01. ***p < .001. The figure presents standardized coefficients. Dashed lines represent non‐significant effects. The value between the main predictor and the dependent variable indicates the direct effect. We controlled for age, gender and political orientation in the analysis.

In the case of vaccine‐related conspiracy beliefs, political anger was the significant positive predictor (*B* = 0.11, SE = 0.05, *p* = .019). The effect of lack of control (*B* = 0.002, SE = 0.07, *p* = .974) was not significant. Consistent with this, the indirect effect through political anger was significant (IE = −0.02, SE_boot_ = 0.01, 95% CI [−0.05, −0.003]), while the indirect effect via lack of control (IE = −0.001, SE_boot_ = 0.02, 95% CI [−0.03, 0.03]) was not significant. The direct effect (*B* = −0.20, SE = 0.07, *p* = .004) remained significant after accounting for the effects of mediators, as was the total effect (*B* = −0.23, *β* = −.13, SE = 0.07, *p* < .001). In this model, political orientation (*B* = 0.49, SE = 0.05, *p* < .001) and age (*B* = 0.01, SE = 0.005, *p* = .036) positively predicted vaccine‐related conspiracy beliefs. Meanwhile, gender had a significant negative effect (*B* = −0.52, SE = 0.15, *p* < .001) on this variable. Figure 8 shows a summary of the model with standardized coefficients.

#### Conspiracy beliefs about financial crises

**FIGURE 9:**
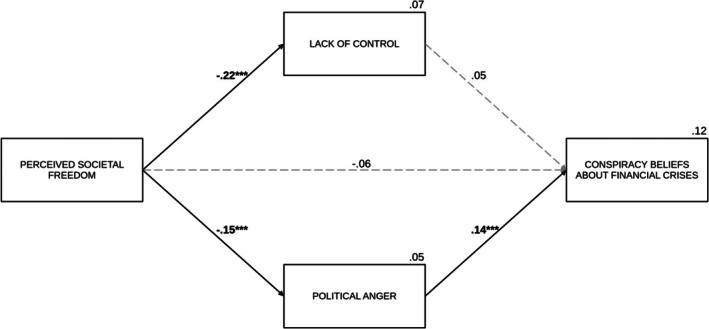
The effect of perceived societal freedom on conspiracy beliefs about financial crises via lack of control and political anger. ***p < .001. The figure presents standardized coefficients. Dashed lines represent non‐significant effects. The value between the main predictor and the dependent variable indicates the direct effect. We controlled for age, gender and political orientation in the analysis.

Political anger (*B* = 0.14, SE = 0.04, *p* < .001) was a significant predictor of conspiracy beliefs about financial crises. However, the effect of lack of control was not significant (*B* = 0.07, SE = 0.06, *p* = .238). This pattern was reflected in a significant indirect effect via political anger (IE = −0.03, SE_boot_ = 0.01, 95% CI [−0.06, −0.01]). In contrast, the indirect effect through the lack of control was not significant (IE = −0.02, SE_boot_ = 0.01, 95% CI [−0.05, 0.01]). The direct effect was not significant this time (*B* = −0.09, SE = 0.06, *p* = .127), while the total effect was significant (*B* = −0.13, *β* = −.10, SE = 0.06, *p* = .020), suggesting a complete mediation. Political orientation (*B* = 0.28, SE = 0.04, *p* < .001) and age (*B* = 0.01, SE = 0.004, *p* = .008) positively predicted conspiracy beliefs about financial crises, while the effect of gender was not significant (*B* = −0.07, SE = 0.12, *p* = .575). For a summary of the model with standardized coefficients, see Figure 9.

### Discussion

Study 5 provided initial evidence of the psychological mechanisms underlying the relationship between perceived societal freedom and conspiracy beliefs. The results revealed that political anger consistently mediated the relationship across all conspiracy belief types, only partially supporting our hypotheses H2a–H2d. Notably, although perceived societal freedom significantly predicted feelings of lack of control, lack of control itself did not predict any of the examined conspiracy beliefs. This finding is consistent with recent meta‐analytic evidence by Stojanov and Halberstadt (2020), who found minimal support for the widely assumed relationship between a lack of control and conspiracy beliefs in the experimental studies. Together, these results suggest that conspiracy beliefs in response to restricted societal freedom are driven by emotional responses (Harmon‐Jones & Szymaniak, 2023; Szymaniak et al., 2023) to perceived constraints. However, since mediations were not complete in several models, future research should explore additional psychological mechanisms underlying this relationship. Furthermore, they should provide evidence on causality in the full mediation paths.

## GENERAL DISCUSSION

The present research examined the relationship between societal freedom and conspiracy beliefs through five complementary studies that tested how both objectively measured societal freedom and individual perceptions of it are related to reduced beliefs in generic and content‐specific conspiracy theories. Studies 1 and 2 supported our hypotheses providing evidence for the negative relationship between country‐level societal freedom, as measured by the Human Freedom Index (HFI) and conspiracy beliefs. Specifically, in Study 1, we found that higher levels of HFI were associated with lower levels of endorsement of generic conspiracy beliefs. Study 2 extended these findings using a larger dataset and focusing on interest group‐related COVID‐19 conspiracy beliefs, demonstrating that the relationship between HFI and reduced conspiracy beliefs holds even when examining conspiracies that do not explicitly refer to government actors. Moving from the country level to the individual level, Study 3 found that higher perceived societal freedom was associated with lower endorsement of several types of conspiracy beliefs, including conspiracy mentality, vaccine‐related conspiracies and beliefs about financial crises. Study 4 provided causal evidence that manipulating perceptions of societal freedom influenced conspiracy belief endorsement, with participants in the low‐freedom condition endorsing higher levels of conspiracy beliefs compared to those in the high‐freedom and control conditions. Finally, Study 5 examined the psychological mechanisms underlying this relationship, revealing that political anger served as a mediator of the relationship between perceived societal freedom and conspiracy beliefs.

In our theoretical framework, we proposed that low and high societal freedom may increase or decrease conspiracy beliefs, respectively, through similar mechanisms that operate in opposite directions. The mediation analyses revealed that political anger serves as a consistent mechanism explaining this relationship across all conspiracy belief types examined. This reflects the findings that restrictions on freedom evoke anger when individuals perceive their autonomy as constrained (Brehm, 1966; Dillard & Shen, 2005) and this anger subsequently creates an emotional readiness to attribute negative events to malevolent actors, facilitating conspiracy belief endorsement (Harmon‐Jones & Szymaniak, 2023; Szymaniak et al., 2023). Notably, while perceived societal freedom predicted increased feelings of lack of control, this sense of lack of control did not have an effect on conspiracy beliefs. This finding aligns with recent meta‐analytic work demonstrating weak empirical support in the experimental studies for this widely theoretically discussed relationship (Stojanov & Halberstadt, 2020). Importantly, there are previous studies (e.g., Gillespie & Ghumkhor, 2024; Pinazo‐Calatayud et al., 2024) that theoretically and empirically considered freedom restrictions as part of a conspiracy theory related to the pandemic and the hidden purposes of powerful groups for the control management of the people. Therefore, in our studies, we examined various types of conspiracy beliefs spanning different domains, including also vaccine‐related conspiracies unrelated directly to freedom restrictions, thereby demonstrating that the relationship between societal freedom and conspiratorial thinking extends beyond government‐focused narratives. This broad pattern suggests that the observed relationship reflects a general tendency rather than being limited to conspiracies explicitly involving themes of control or restriction of freedom.

Overall, our research advances the emerging literature on macro‐level predictors of conspiracy beliefs (Hornsey, Bierwiaczonek, et al., 2023) by establishing societal freedom—both institutionally embedded and individually perceived—as a protective factor against such beliefs. This emphasis on macro‐level factors that can lead to beliefs in conspiracy theories represents a theoretical advancement beyond the traditional focus on individual psychological tendencies such as uncertainty (van Prooijen & Jostmann, 2013) that account for increases in people's beliefs in conspiracy theories. While Hornsey, Pearson, et al. (2023) demonstrate that conspiracy beliefs proliferate in nations experiencing economic hardship, our research extends this perspective by examining how societal environments characterized by limited civil liberties and institutional transparency shape individuals' propensity to adopt conspiracy beliefs. This perspective also aligns with research showing that conspiracy beliefs are responses to environmental cues about e.g. corruption (Alper, 2023; Alper & Imhoff, 2023). Together, our findings contribute to theoretical frameworks that view conspiracy beliefs as tools to defend one's beliefs (van Prooijen, 2022) by demonstrating the role which restricted societal freedom may play in shaping the endorsement of such beliefs.

### Limitations and future directions

While the present studies provide robust, multi‐design evidence for the relationship between societal freedom and conspiracy beliefs, several limitations suggest avenues for further investigation. Studies 1, 2, 3 and 5 were correlational, limiting causal claims. Although Study 4 directly manipulated perceived societal freedom, future work could benefit from longitudinal designs, particularly focusing on freedom at the country level. Longitudinal designs can provide more robust evidence about the consistency of the direction of the effects of societal freedom on conspiracy beliefs, because freedom can evolve and change over time. For that reason, a potential avenue for future research is to empirically test and compare the effects of societal freedom on conspiracy beliefs in contexts where societal freedom is (vs. is not) at risk of being restricted. In addition, assessing changes in conspiracy beliefs as a country transitions from a more authoritarian regime to a more democratic one (or vice versa) would shed light on whether increased (or decreased) societal freedoms alter adopting conspiratorial explanations over time. We already know from the literature that authoritarian regimes can act as catalysts for people's conspiratorial explanations of events (e.g., Giry & Gürpınar, 2020), therefore, comparative evidence from contexts experiencing political transition would be highly informative. Moreover, an important consideration for future research is whether the effects of subjective freedom are relative or absolute. In Study 4, our experimental manipulation explicitly asked participants to compare their situations with people in other countries, effectively enhancing the salience of freedom restrictions (or lack thereof) at the societal level. This raises the question of whether the observed effects depend on such comparisons. Future research should examine whether manipulations of perceived freedom, without cross‐national comparisons, produce similar effects on conspiracy beliefs, which would have important implications for both theoretical understanding and intervention design.

Moreover, while Study 4 provided initial causal evidence for the societal freedom–conspiracy relationship, several limitations underscore the need for more comprehensive causal investigation. The non‐significant difference between the low‐freedom and control conditions in the case of conspiracy beliefs about financial crises suggests that our understanding of how perceptions of societal freedom causally influence conspiracy beliefs may be incomplete. This pattern may indicate that different types of conspiracy beliefs are shaped more strongly by domain‐specific freedom restrictions (e.g., perceived economic restrictions would shape conspiracy beliefs about financial crises more strongly) and that this factor should be controlled in experimental manipulations. Alternatively, this pattern may suggest that experimental manipulations more easily influence existing conspiracy beliefs than generate belief in new ones. Moreover, although we identified political anger as a significant mediator in our correlational study, the causal relationships within this full path remain untested. Additionally, the partial mediations observed across several models indicate that our current framework captures only part of the processes linking societal freedom to conspiracy beliefs. Identifying and testing additional mechanisms might be crucial for developing a more comprehensive theoretical account of how societal freedom shapes conspiratorial thinking.

Furthermore, while our research has focused primarily on political and scientific conspiracies, conspiracy theories can manifest in a variety of domains, including natural disasters, cultural events and supernatural phenomena (Douglas & Sutton, 2023). While Studies 3, 4 and 5 demonstrated that the relationship between perceived societal freedom and conspiracy beliefs extends within Poland beyond government‐focused conspiracies to areas such as vaccine‐related conspiracies, the country‐level analyses in Studies 1 and 2 were limited to generic and COVID‐19‐related conspiracy theories. Therefore, we did not have strong evidence as to whether the negative association between societal freedom and conspiracy thinking observed at the country level generalizes to these broader, less institutionally focused conspiracy domains. Future cross‐national research should examine whether country‐level freedom predicts conspiracy theory endorsement in different domains, which could help refine intervention strategies for different types of conspiracy beliefs. Additionally, some items from the scale of conspiracy beliefs about financial crises (van Prooijen et al., 2015) may not clearly indicate conspiratorial thinking because they do not directly imply secret plots and could be interpreted as reasonable concerns about economic crises. Future research should consider developing measures that more precisely capture beliefs about coordinated, secret actions by powerful groups. Moreover, the non‐significant effect observed for generic conspiracy beliefs using Lantian et al.'s (2016) scale in Study 3 underscores the importance of considering the limitations of single‐item measures. Although we found this effect in Study 5, the consistency of the effects observed in the case of Kay and Slovic's (2023) multi‐item Generic Conspiracist Beliefs scale across studies suggests that single‐item measures may be problematic due to their susceptibility to measurement error at the individual level. Notably, this issue did not occur in Study 1, where we operated on country‐level means, which likely reduced individual‐level measurement error through aggregation.

A clear strength of Studies 1 and 2 is their external validity. They have large, diverse samples which can be coupled with objective indicators of national contexts. However, cross‐national datasets also have field‐wide constraints. Many country‐level indicators that are likely to be related to belief in conspiracies (e.g., freedom and corruption) are highly correlated (see e.g. Toribio‐Flórez et al., 2025). This makes it difficult to identify unique drivers using standard country‐level controls, as incorporating several collinear level‐2 predictors can result in yielding unstable estimates and biased standard errors (Clark, 2013, see also Can et al., 2014; Yaremych & Preacher, 2024). Additionally, country‐level analyses raise the caution that relationships among country means should not be assumed to hold for individuals (the ecological fallacy; Robinson, 1950). The subsequent studies we conducted helped to address these shortcomings. Study 3 examined the same link at the individual level, showing that those who perceive greater societal freedom report lower endorsement of various conspiracy beliefs. Study 4 manipulated perceived societal freedom and observed corresponding changes in conspiracy beliefs, providing initial causal evidence at the individual level, while Study 5 found the variable that may mediate this relationship. Together, the pattern across these three studies suggests that the country‐level association is rather not merely a by‐product of omitted collinear variables.

Lastly, as it was earlier mentioned in the introduction, we proposed that conspiracy beliefs constitute a form of beliefs‐based reaction against the freedom restrictive policies. One potentially interesting and informative avenue for future research will be to extend this argument to the forms of collective action and support for collective action forms that emerge from the endorsement of different types of conspiracy theories: generic and content‐specific (see also Thomas et al., 2024). If such effects can be observed especially after experimentally manipulating freedom restrictions, then causal evidence can provide useful and robust insights on the negative consequences of freedom restrictive policies.

#### Policy implications

The present research has important implications for policymakers seeking to reduce the spread of conspiracy beliefs. Across five studies, we demonstrated that objective societal freedom, measured at the country level and subjective perceptions of societal freedom, measured at the individual level, are both negatively associated with conspiracy beliefs. This dual evidence suggests that conspiracy thinking can be reduced not only by guaranteeing civil liberties and transparency structurally but also by enhancing individuals' perceptions of autonomy. However, we propose that these two dimensions should be addressed sequentially, with structural foundations providing the precondition for successful individual‐level interventions.

At the structural level, policies that secure pluralistic media, legal safeguards and opportunities for civic participation are critical. These measures strengthen objective freedom by ensuring access to diverse information, increasing government accountability and reducing distrust of authorities. These institutional protections are especially vital in times of crisis when restrictions on freedom can foster distrust and conspiracy narratives. Based on our individual‐level results, we suggest that communication strategies during public crises should emphasize autonomy‐supportive framing and clarify how regulations protect public welfare while preserving individual choice. However, we postulate that such interventions are only effective at the individual level when the structural freedoms are present. Importantly, we caution against using these communication strategies in contexts where objective freedoms are severely limited, such as under authoritarian regimes. Attempting to convince individuals living under genuine restrictions that they have choices and autonomy could appear disconnected from reality and backfire by increasing rather than decreasing conspiracy beliefs. Therefore, policymakers should view freedom as both a legal‐institutional asset and a psychological resource that requires authentic structural foundations before it can be effectively communicated and serve as a buffer against conspiracy beliefs.

## AUTHOR CONTRIBUTIONS

**Maciej Siemiątkowski:** Conceptualization; investigation; writing – original draft; methodology; writing – review and editing; visualization; formal analysis; project administration; data curation; resources. **Theofilos Gkinopoulos:** Conceptualization; investigation; funding acquisition; writing – original draft; writing – review and editing; methodology; supervision; resources. **Michał Bilewicz:** Resources; supervision; writing – review and editing; writing – original draft; conceptualization; investigation; funding acquisition; methodology.

## FUNDING INFORMATION

The work has been supported by a grant from the Priority Research Area (Future Society: Behaviour in Crisis Lab ‐ Flagship Project) under the Strategic Programme Excellence Initiative at Jagiellonian University. Grant no: U1U/P02/NO/21.97 awarded to the second author, and a National Science Center (NCN) Opus grant (Grant no: DEC‐2023/49/B/HS6/01428) awarded to the third author.

## CONFLICT OF INTEREST

The authors report no conflict of interest.

## Supporting information


Data S1.

## Data Availability

The data and analysis scripts for all studies have been made publicly available via OSF and can be accessed at https://osf.io/dkczs/.

## Footnotes

^1^
In both studies that used the HFI index, we calculated models with HFI as the sole predictor of conspiracy beliefs (see the Data S1). The results of these analyses were consistent with those presented in the main text.

^2^
Following a suggestion from one of the reviewers, for all analyses that included political orientation, we also added its squared term. These results are presented in the Data S1 and show that adding this term does not generally change our conclusions, except for one comparison in Study 4 that became non‐significant.
