# Supplementary material for: Free from conspiracies: The negative relationship between societal freedom and belief in generic and content‐specific conspiracy theories
Source: Br J Soc Psychol. 2025 Nov 24;65(1):e70021. doi: 10.1111/bjso.70021 (PMC12641589; doi:10.1111/bjso.70021)
Supplement: Supplementary file 1 — Data S1. [file BJSO-65-0-s001.docx]

Supplementary materials

**Supplementary analyses on data from Study 1**

Table S1

*Means, standard deviations, and correlations for individual-level variables used in Study 1*

| Variable | *M* | *SD* | 1 | 2 |
| --- | --- | --- | --- | --- |
|  |  |  |  |  |
| 1. Generic conspiracy beliefs | 5.97 | 2.03 |  |  |
|  |  |  |  |  |
| 2. Age | 21.81 | 5.62 | .01 |  |
|  |  |  |  |  |
| 3. Gender | 0.66 | 0.48 | .0004 | -.02 |
|  |  |  |  |  |

*Note.* Gender was a dichotomous variable with values of 0 = man and 1 = woman.

The descriptive statistics for the Human Freedom Index (the only country-level variable) were as follows: *M* = 7.65; *SD* = 0.92.

***Results of the model without individual-level covariates***

As recommended by one of the reviewers, we conducted analyses from this study without individual-level covariates. We compared the baseline model (Model 0) with a model that included the HFI as the only country-level predictor (Table S2). This comparison revealed a significant improvement in model fit (χ²(1) = 7.49, *p* = .006). The HFI was found to be a significant negative predictor of generic conspiracy beliefs (β = -.13) and the effect was almost identical to the result obtained in the analysis with covariates (β = -.14).

Table S2

*Human Freedom Index as an only predictor of generic conspiracy beliefs*

|  | Model 0 | | Model 1 | |
| --- | --- | --- | --- | --- |
| Predictors | *B (SE)* | *p* | *B (SE)* | *p* |
| (intercept) | 5.98 (0.10) | <.001 | 5.98 (0.09) | <.001 |
| **Country-level effects** |  |  |  |  |
| Human Freedom Index (HFI) |  |  | -0.29 (0.10) | .008 |
| **Random effects** |  |  |  |  |
| σ^2^ | 3.80 |  | 3.80 |  |
| *τ_00_* | 0.35 _Country_ |  | 0.28 _Country_ |  |
| ICC | .08 |  | .07 |  |
| *N* (countries) | 36 |  | 36 |  |
| Observations | 6353 |  | 6353 |  |
| Marginal *R^2^* | .00 |  | .02 |  |
| Conditional *R^2^* | .08 |  | .09 |  |
| Deviance | 26599 |  | 26592 |  |

**Supplementary analyses on data from Study 2**

Table S3

*Means, standard deviations, and correlations for individual-level variables used in Study 2*

| Variable | *M* | *SD* | 1 | 2 | 3 |
| --- | --- | --- | --- | --- | --- |
|  |  |  |  |  |  |
| 1. Interest groups-related COVID-19 conspiracy beliefs | 2.61 | 3.16 |  |  |  |
|  |  |  |  |  |  |
| 2. Age | 43.23 | 16.10 | -.09*** |  |  |
|  |  |  |  |  |  |
| 3. Gender | 0.52 | 0.50 | .002 | -.09*** |  |
|  |  |  |  |  |  |
| 4. Political orientation | 4.95 | 2.32 | .19*** | .02*** | -.08*** |
|  |  |  |  |  |  |

*Note.* *** indicates *p* < .001. Gender was a dichotomous variable with values of 0 = man and 1 = woman. The descriptive statistics for the Human Freedom Index (the only country-level variable) were as follows: *M* = 7.57; *SD* = 1.00.

***Results of the model without individual-level covariates***

As in Study 1, we conducted analyses without individual-level covariates (Table S4). The comparison between the baseline model and a model that included only the HFI showed a significant improvement in model fit (χ²(1) = 14.14, *p* < .001). The HFI remained a significant negative predictor of interest groups-related COVID-19 conspiracy beliefs (β = -.18), with the effect being almost identical to the one in the main analysis with covariates (β = -.17).

Table S4

*HFI as an only predictor of interest groups-related COVID-19 conspiracy beliefs*

|  | Model 0 | | Model 1 | |
| --- | --- | --- | --- | --- |
| Predictors | *B (SE)* | *p* | *B (SE)* | *p* |
| (intercept) | 2.74 (0.17) | <.001 | 2.65 (0.16) | <.001 |
| **Country-level effects** |  |  |  |  |
| Human Freedom Index (HFI) |  |  | -0.62 (0.16) | <.001 |
| **Random effects** |  |  |  |  |
| σ^2^ | 8.52 |  | 8.52 |  |
| *τ_00_* | 1.58 _Country_ |  | 1.22 _Country_ |  |
| ICC | .16 |  | .13 |  |
| *N* (countries) | 52 |  | 52 |  |
| Observations | 44458 |  | 44458 |  |
| Marginal *R^2^* | .00 |  | .03 |  |
| Conditional *R^2^* | .16 |  | .15 |  |
| Deviance | 221649 |  | 221635 |  |

**Results of the model with an additional squared term for political orientation**

In line with reviewer's suggestion that curvilinear relationships of political orientation with conspiracy beliefs may be more robust than linear ones (Imhoff et al., 2022), we conducted additional analyses incorporating both linear and quadratic terms for political orientation (Table S5). Model 1 significantly improved the fit compared to the baseline model (χ²(4) = 1349.42, *p* < .001). The linear (β = .15) and quadratic (β = .05) terms for political orientation were both significant predictors of belief in interest groups-related COVID-19 conspiracy beliefs, while the effect of age remained negative (β = -.04) and the effect of gender remained non-significant (β = .01). Adding the HFI in Model 2 resulted in a significant improvement in model fit (χ²(1) = 13.52, *p* < .001). Crucially, the HFI remained a significant negative predictor (β = -.17), with a virtually identical effect to that found in our main analysis (β = -.17).

Table S5

*Individual and country-level predictors of interest groups-related COVID-19 conspiracy beliefs*

|  | Model 0 | | Model 1 | | Model 2 | |
| --- | --- | --- | --- | --- | --- | --- |
| Predictors | *B (SE)* | *p* | *B (SE)* | *p* | *B (SE)* | *p* |
| (intercept) | 2.74 (0.17) | <.001 | 2.59 (0.17) | <.001 | 2.50 (0.15) | <.001 |
| **Individual-level effects** |  |  |  |  |  |  |
| Age |  |  | -0.01 (0.001) | <.001 | -0.01 (0.001) | <.001 |
| Gender |  |  | 0.04 (0.03) | .128 | 0.04 (0.03) | .127 |
| Political orientation |  |  | 0.21 (0.01) | <.001 | 0.21 (0.01) | <.001 |
| Political orientation squared |  |  | 0.02 (0.002) | <.001 | 0.02 (0.002) | <.001 |
| **Country-level effects** |  |  |  |  |  |  |
| Human Freedom Index (HFI) |  |  |  |  | -0.57 (0.15) | <.001 |
| **Random effects** |  |  |  |  |  |  |
| σ^2^ | 8.52 |  | 8.26 |  | 8.26 |  |
| *τ_00_* | 1.58 _Country_ |  | 1.39 _Country_ |  | 1.09 _Country_ |  |
| ICC | .16 |  | .14 |  | .12 |  |
| *N* (countries) | 52 |  | 52 |  | 52 |  |
| Observations | 44458 |  | 44458 |  | 44458 |  |
| Marginal *R^2^* | .00 |  | .03 |  | .06 |  |
| Conditional *R^2^* | .16 |  | .17 |  | .17 |  |
| Deviance | 221649 |  | 220300 |  | 220286 |  |

*Note*. Gender was a dichotomous variable with values of 0 = man and 1 = woman.

**Analyses from Study 2 using the full COVID-19 conspiracy belief scale as the DV**

**COVID-19 conspiracy beliefs** were measured using a four-item scale assessing the extent to which participants believed that e.g. “The coronavirus (COVID-19) is a hoax invented by interest groups for financial gains” (α = .92). Responses were provided on an 11-point scale (0 = strongly disagree, 10 = strongly agree).

**Results**

Model 1 included individual-level predictors (age, gender, and political ideology) and significantly improved model fit compared to Model 0, χ²(3) = 1902.20, *p* < .001. In contrast to the main analysis, gender emerged as a significant positive predictor of conspiracy beliefs (β = .03). Age maintained a negative association with conspiracy beliefs (β = -.03), and political orientation showed a significant positive effect (β = .19). In Model 2, the Human Freedom Index (HFI) was introduced as the country-level predictor, which significantly improved the model fit compared to Model 1, χ²(1) = 10.53, *p* = .001. Consistent with the main analysis, HFI showed a significant negative relationship with conspiracy beliefs (β = -.16). The effects of individual-level predictors remained stable across models (Table S6).

Table S6

*Individual-level and country-level predictors of COVID-19 conspiracy beliefs*

|  | Model 0 | | Model 1 | | Model 2 | |
| --- | --- | --- | --- | --- | --- | --- |
| Predictors | *B (SE)* | *p* | *B (SE)* | *p* | *B (SE)* | *p* |
| (intercept) | 3.17 (0.17) | <.001 | 3.09 (0.16) | <.001 | 3.01 (0.15) | <.001 |
| **Individual-level effects** |  |  |  |  |  |  |
| Age |  |  | -0.005 (0.001) | <.001 | -0.005 (0.001) | <.001 |
| Gender |  |  | 0.16 (0.03) | <.001 | 0.16 (0.03) | <.001 |
| Political orientation |  |  | 0.24 (0.01) | <.001 | 0.24 (0.01) | <.001 |
| **Country-level effects** |  |  |  |  |  |  |
| Human Freedom Index (HFI) |  |  |  |  | -0.50 (0.15) | .002 |
| **Random effects** |  |  |  |  |  |  |
| σ^2^ | 7.08 |  | 6.79 |  | 6.79 |  |
| *τ_00_* | 1.52 _Country_ |  | 1.32 _Country_ |  | 1.10 _Country_ |  |
| ICC | .18 |  | .16 |  | .14 |  |
| *N* (countries) | 52 |  | 52 |  | 52 |  |
| Observations | 44458 |  | 44458 |  | 44458 |  |
| Marginal *R^2^* | .00 |  | .04 |  | .07 |  |
| Conditional *R^2^* | .18 |  | .19 |  | .20 |  |
| Deviance | 213463 |  | 211560 |  | 211550 |  |

*Note*. Gender was a dichotomous variable with values of 0 = man and 1 = woman.

**Perceived societal freedom scale**

We prepared this scale based on indicators used to create the Human Freedom Index. The scale includes eight items assessing perceptions of different domains of freedom: rule of law, security and safety, movement, religion, association and assembly, expression and information, relationships, and economic freedom. Each item is rated on a scale from 1 (not at all) to 7 (very much so).

Different countries have varying levels of freedom in different domains of people's lives.We are interested in understanding how you perceive your country in relation to these different domains. Please read each of the following items carefully and answer to what extent in your opinion they characterize Poland using a scale ranging from 1 - not at all to 7 - very much so.

Rule of law

The rule of law is an essential condition of freedom that protects the individual from coercion by others. It means that everyone, including the government, must follow established laws and procedures. Strong rule of law prevents exposure to abuse by the authorities and submission to others’ will.

Security and safety

Security and safety refer to protection from violence, crime, and threats to personal well-being. High security and safety promote the right to life and to safety from physical aggressions and transgressions that can reduce or eliminate personal freedom.

Movement

Freedom of movement includes the right to travel within one's country, leave and return to it, and choose where to live. Movement defines this basic human right for a free society and governments that restrict people’s movement greatly limit the scope of overall liberty.

Religion

Religion component refers to the respect by free societies towards the right to religious practices of one’s choosing. Religious freedom protects the right to practice and choose one’s religion, to proselytize peacefully, and to change religions.

Association, assembly, and civil society

This component refers to the freedom in civil societies to associate and assemble with peaceful individuals or organizations of one’s choice, to carry out peaceful protests and to form or join organizations for social, political etc. purposes.

Expression and information

This component reflects a broad range of freedoms, such as personal expression, the press, use of the internet and media platforms, as well as lack of murders, harassment or punishment of journalists, who freely express their opinions.

Relationships

Relationships component refers to freedoms to have intimate and familial relationships with others, with those relationships being based on equal rights, as well as the freedom to establish a relationship with a person of your choice.

Economic freedom

It describes the fundamental right of humans to control their own labor and property. It also relates to being free to work, produce, consume, and invest in any way they please, as well as the governments’ permission to free movement of labor, capital, and goods.

**Conspiracy beliefs about financial crises scale**

From time to time countries across the globe experience financial crises which widely affect the economic life of citizens and the infrastructures of those countries affected by such financial downturns. Please, read the statements below regarding the origins, reasons or consequences of financial crises and rate your agreement using a scale from 1 = strongly disagree to 7 = strongly agree.

1. Financial crises are caused deliberately.
2. Governments profit from financial crises in ways that most people are unaware of.
3. Financial crises are the result of a conspiracy between bankers and corrupt politicians.
4. Financial crises have been caused by some banks to win the competition from other banks.
5. A group of powerful individuals had a vested interest in past financial crises.
6. Past financial crises mainly served to increase the wealth of a limited number of individuals.

**Supplementary analyses on data from Study 3**

Table S7

*Means, standard deviations, and correlations for variables used in Study 3*

| Variable | *M* | *SD* | 1 | 2 | 3 | 4 | 5 | 6 | 7 | 8 |
| --- | --- | --- | --- | --- | --- | --- | --- | --- | --- | --- |
|  |  |  |  |  |  |  |  |  |  |  |
| 1. Perceived societal freedom | 5.03 | 1.15 |  |  |  |  |  |  |  |  |
|  |  |  |  |  |  |  |  |  |  |  |
| 2. Generic conspiracy beliefs | 4.94 | 1.64 | -.09 |  |  |  |  |  |  |  |
|  |  |  |  |  |  |  |  |  |  |  |
| 3. Conspiracy mentality | 67.90 | 22.53 | -.17** | .57*** |  |  |  |  |  |  |
|  |  |  |  |  |  |  |  |  |  |  |
| 4. Generic conspiracist beliefs | 3.30 | 1.39 | -.17** | .51*** | .75*** |  |  |  |  |  |
|  |  |  |  |  |  |  |  |  |  |  |
| 5. Vaccine-related conspiracy beliefs | 4.12 | 2.07 | -.21*** | .50*** | .64*** | .67*** |  |  |  |  |
|  |  |  |  |  |  |  |  |  |  |  |
| 6. Conspiracy beliefs about financial crises | 4.44 | 1.62 | -.17** | .52*** | .80*** | .75*** | .65*** |  |  |  |
|  |  |  |  |  |  |  |  |  |  |  |
| 7. Gender | 0.62 | 0.49 | .04 | -.05 | -.15* | -.04 | -.05 | -.04 |  |  |
|  |  |  |  |  |  |  |  |  |  |  |
| 8. Age | 43.63 | 15.84 | .15* | -.04 | -.05 | -.08 | -.04 | -.01 | .20*** |  |
|  |  |  |  |  |  |  |  |  |  |  |
| 9. Political orientation | 4.19 | 1.58 | -.10 | .18** | .32*** | .23*** | .28*** | .29*** | .06 | -.04 |
|  |  |  |  |  |  |  |  |  |  |  |

*Note.* * indicates *p* < .05. ** indicates *p* < .01. *** indicates *p* < .001. Gender was a dichotomous variable with values of 0 = woman and 1 = man.

**Results of the models with an additional squared term for political orientation**

Following the approach used in Study 2, we tested whether including a measure of political orientation in both linear and quadratic forms affected the findings of Study 3.

***Generic conspiracy beliefs***

In Model 1 (Table S8) the effect of the quadratic term of political orientation was not significant (β = .51), similarly as the effects of the linear term (β = -.31), gender (β = -.06) and age (β = -.03). There was no significant improvement in model fit when perceived societal freedom was added in Model 2. This time, the quadratic political orientation term was a significant predictor (β = .54), while the effect of perceived societal freedom was not significant (β = -.08). The effect of the other variables does not change compared to Model 1. These results are consistent with our main analyses.

Table S8

*Hierarchical regression results - Generic Conspiracy Beliefs as the DV*

|  | Model 1 | | | 95% *CI* | | Model 2 | | | 95% *CI* | |
| --- | --- | --- | --- | --- | --- | --- | --- | --- | --- | --- |
|  | *B* | *SE* | *p* | LL | UL | *B* | *SE* | *p* | LL | UL |
| (intercept) | 5.34 | 0.65 | <.001 | 4.06 | 6.61 | 5.96 | 0.79 | <.001 | 4.40 | 7.51 |
| Gender | -0.21 | 0.20 | .316 | -0.61 | 0.20 | -0.20 | 0.20 | .326 | -0.60 | 0.20 |
| Age | -0.003 | 0.01 | .622 | -0.02 | 0.01 | -0.002 | 0.01 | .760 | -0.01 | 0.01 |
| Political orientation | -0.32 | 0.27 | .236 | 0.07 | 0.31 | -0.36 | 0.27 | .189 | -0.90 | 0.18 |
| Political orientation squared | 0.06 | 0.03 | .053 | -0.001 | 0.12 | 0.06 | 0.03 | .042 | 0.002 | 0.13 |
| Perceived societal freedom |  |  |  |  |  | -0.12 | 0.09 | .175 | -0.29 | 0.05 |
| *R^2^* | .05 | |  |  |  | .06 | |  |  |  |
| *F* | 3.55 | | .008 |  |  | 3.22 | | .008 |  |  |
| Δ*R^2^* |  | |  |  |  | .01 | |  |  |  |
| Δ*F* |  | |  |  |  | 1.85 | | .175 |  |  |

***Conspiracy Mentality***

Results of Model 1 (Table S9) showed that gender was a significant predictor, with men reporting lower conspiracy mentality (β = -.18). The effect of the quadratic political orientation term was also significant (β = .66), while age (β = -.01) and the linear political orientation term (β = -.31) did not significantly predict conspiracy mentality. The addition of perceived societal freedom in Model 2 resulted in a significant improvement in model fit. Perceived societal freedom emerged as a significant negative predictor (β = -0.15), consistent with our main findings. The effects of the covariates remained stable across models.

Table S9

*Hierarchical regression results - Conspiracy Mentality as the DV*

|  | Model 1 | | | 95% *CI* | | Model 2 | | | 95% *CI* | |
| --- | --- | --- | --- | --- | --- | --- | --- | --- | --- | --- |
|  | *B* | *SE* | *p* | LL | UL | *B* | *SE* | *p* | LL | UL |
| (intercept) | 70.81 | 8.37 | <.001 | 54.32 | 87.29 | 86.09 | 10.12 | <.001 | 66.17 | 106.02 |
| Gender | -8.48 | 2.64 | .001 | -13.67 | -3.28 | -8.37 | 2.61 | .002 | -13.51 | -3.23 |
| Age | -0.02 | 0.08 | .826 | -0.18 | 0.14 | 0.01 | 0.08 | .891 | -0.15 | 0.17 |
| Political orientation | -4.48 | 3.52 | .204 | -11.42 | 2.45- | -5.39 | 3.50 | .125 | -12.29 | 1.50 |
| Political orientation squared | 1.09 | 0.41 | .008 | 0.29 | 1.89 | 1.18 | 0.40 | .004 | 0.38 | 1.20 |
| Perceived societal freedom |  |  |  |  |  | -2.88 | 1.10 | .009 | -5.04 | -0.72 |
| *R^2^* | .16 | |  |  |  | .18 | |  |  |  |
| *F* | 12.54 | | <.001 |  |  | 11.63 | | <.001 |  |  |
| Δ*R^2^* |  | |  |  |  | .02 | |  |  |  |
| Δ*F* |  | |  |  |  | 6.91 | | .009 |  |  |

***Generic conspiracist beliefs***

In the case of Model 1 (Table S10) none of the individual predictors reached statistical significance: gender (β = -.05), age (β = -.07), linear political orientation (β = -.004) and quadratic political orientation (β = .24). When perceived societal freedom was added to Model 2, there was a significant improvement in model's fit. Perceived societal freedom emerged as a significant negative predictor (β = -.15), which is consistent with the results of the main analyses (β = -.14). The effects of the covariates remained stable across models.

Table S10

*Hierarchical regression results - Generic Conspiracist Beliefs as the DV*

|  | Model 1 | | | 95% *CI* | | Model 2 | | | 95% *CI* | |
| --- | --- | --- | --- | --- | --- | --- | --- | --- | --- | --- |
|  | *B* | *SE* | *p* | LL | UL | *B* | *SE* | *p* | LL | UL |
| (intercept) | 3.19 | 0.54 | <.001 | 2.12 | 4.27 | 4.15 | 0.66 | <.001 | 2.85 | 5.44 |
| Gender | -0.13 | 0.17 | .453 | -0.47 | 0.21 | -0.12 | 0.17 | .472 | -0.46 | 0.2` |
| Age | -0.01 | 0.01 | .224 | -0.02 | 0.004 | -0.01 | 0.01 | .381 | -0.02 | 0.01 |
| Political orientation | -0.004 | 0.23 | .987 | -0.46 | 0.45 | -0.06 | 0.23 | .791 | -0.58 | 0.44 |
| Political orientation squared | 0.02 | 0.03 | .359 | -0.03 | 0.08 | 0.36 | 0.03 | .263 | -0.02 | 0.08 |
| Perceived societal freedom |  |  |  |  |  | -0.18 | 0.07 | .013 | -0.32 | -0.04 |
| *R^2^* | .06 | |  |  |  | .08 | |  |  |  |
| *F* | 4.54 | | .001 |  |  | 4.96 | | <.001 |  |  |
| Δ*R^2^* |  | |  |  |  | .02 | |  |  |  |
| Δ*F* |  | |  |  |  | 6.32 | | .013 |  |  |

***Vaccine-related conspiracy beliefs***

Although Model 1 showed a significantly better fit than the model without predictors (Table S11), none of the variables reached statistical significance: gender (β = -07), age (β = -.02), linear political orientation (β = .03) and quadratic political orientation (β = .27). Model 2, which included perceived societal freedom, achieved a significantly better fit. Perceived societal freedom emerged as a significant negative predictor (β = -.19) of vaccine-related conspiracy beliefs, which is consistent with the results of the main analyses (β = -.18). The effects of the covariates remained stable across models.

Table S11

*Hierarchical regression results - Vaccine-Related Conspiracy Beliefs as the DV*

|  | Model 1 | | | 95% *CI* | | Model 2 | | | 95% *CI* | |
| --- | --- | --- | --- | --- | --- | --- | --- | --- | --- | --- |
|  | *B* | *SE* | *p* | LL | UL | *B* | *SE* | *p* | LL | UL |
| (intercept) | 3.45 | 0.80 | <.001 | 1.88 | 5.02 | 5.26 | 0.96 | <.001 | 3.37 | 7.15 |
| Gender | -0.30 | 0.25 | .238 | -0.79 | 0.20 | -0.29 | 0.25 | .250 | -0.77 | 0.20 |
| Age | -0.002 | 0.01 | .755 | -0.02 | 0.01 | 0.001 | 0.01 | .896 | -0.01 | 0.02 |
| Political orientation | 0.04 | 0.34 | .914 | -0.63 | 0.70 | -0.07 | 0.33 | .830 | -0.73 | 0.58 |
| Political orientation squared | 0.04 | 0.04 | .300 | -0.04 | 0.11 | 0.30 | 0.05 | .190 | -0.03 | 0.13 |
| Perceived societal freedom |  |  |  |  |  | -0.34 | 0.10 | .001 | -0.55 | -0.14 |
| *R^2^* | .09 | |  |  |  | .12 | |  |  |  |
| *F* | 6.63 | | <.001 |  |  | 7.65 | | <.001 |  |  |
| Δ*R^2^* |  | |  |  |  | .03 | |  |  |  |
| Δ*F* |  | |  |  |  | 10.77 | | .001 |  |  |

***Conspiracy beliefs about financial crises***

For conspiracy beliefs about financial crises (Table S12), the quadratic political orientation term was the only significant predictor in Model 1 (β = .69), with gender (β = -.08), age (β = .002) and linear political orientation (β = -.38) showing no significant effects. The addition of perceived societal freedom significantly improved the model's fit. This variable was found to be a negative predictor (β = -.16) of conspiracy beliefs about financial crises, which matched our main findings (β = -.14). The effects of all covariates in Model 2 remained mostly unchanged.

Table S12

*Hierarchical regression results - Conspiracy beliefs about financial crises as the DV*

|  | Model 1 | | | 95% *CI* | | Model 2 | | | 95% *CI* | |
| --- | --- | --- | --- | --- | --- | --- | --- | --- | --- | --- |
|  | *B* | *SE* | *p* | LL | UL | *B* | *SE* | *p* | LL | UL |
| (intercept) | 4.60 | 0.62 | <.001 | 3.38 | 5.82 | 5.76 | 0.75 | <.001 | 4.29 | 7.23 |
| Gender | -0.25 | 0.20 | .196 | -0.64 | 0.13 | -0.25 | 0.19 | .205 | -0.62 | 0.13 |
| Age | 0.0002 | 0.01 | .976 | -0.01 | 0.01 | 0.002 | 0.01 | .692 | -0.01 | 0.01 |
| Political orientation | -0.39 | 0.26 | .132 | -0.90 | 0.12 | -0.46 | 0.26 | .075 | -0.97 | 0.05 |
| Political orientation squared | 0.08 | 0.03 | .007 | 0.02 | 0.14 | 0.09 | 0.03 | .003 | 0.03 | 0.15 |
| Perceived societal freedom |  |  |  |  |  | -0.22 | 0.08 | .007 | -0.38 | -0.06 |
| *R^2^* | .11 | |  |  |  | .13 | |  |  |  |
| *F* | 8.40 | | <.001 |  |  | 8.33 | | <.001 |  |  |
| Δ*R^2^* |  | |  |  |  | .02 | |  |  |  |
| Δ*F* |  | |  |  |  | 7.28 | | .007 |  |  |

**Supplementary analyses on data from Study 4**

Table S13

*Means, standard deviations, and correlations for variables used in Study 4*

| Variable | *M* | *SD* | 1 | 2 | 3 | 4 | 5 |
| --- | --- | --- | --- | --- | --- | --- | --- |
|  |  |  |  |  |  |  |  |
| 1. Generic conspiracist beliefs | 3.17 | 1.09 |  |  |  |  |  |
|  |  |  |  |  |  |  |  |
| 2. Conspiracy beliefs about financial crises | 4.20 | 1.49 | .71*** |  |  |  |  |
|  |  |  |  |  |  |  |  |
| 3. Vaccine-related conspiracy beliefs | 3.59 | 1.93 | .59*** | .54*** |  |  |  |
|  |  |  |  |  |  |  |  |
| 4. Political orientation | 3.96 | 1.54 | .33*** | .35*** | .36*** |  |  |
|  |  |  |  |  |  |  |  |
| 5. Age | 41.66 | 17.15 | .01 | .19** | .13* | .03 |  |
|  |  |  |  |  |  |  |  |
| 6. Gender | 0.47 | 0.50 | .02 | .02 | -.04 | .13* | -.08 |
|  |  |  |  |  |  |  |  |

*Note.* * indicates *p* < .05. ** indicates *p* < .01. *** indicates *p* < .001. Gender was a dichotomous variable with values of 0 = woman and 1 = man.

Table S14

*Fixed-Effects ANCOVA results - Generic conspiracist beliefs as the DV*

| Predictor | Sum  of  Squares | *df* | Mean  Square | *F* | *p* | _partial_ η^2^ | _partial_ η^2^  90% CI  [LL, UL] |
| --- | --- | --- | --- | --- | --- | --- | --- |
| (Intercept) | 115.46 | 1 | 115.46 | 119.52 | < .001 |  |  |
| Condition | 28.09 | 2 | 14.04 | 14.54 | < .001 | .11 | [.05, .17] |
| Gender | 0.03 | 1 | 0.03 | 0.03 | .865 | .00 | [.00, .01] |
| Age | 0.19 | 1 | 0.19 | 0.20 | .654 | .00 | [.00, .02] |
| Political orientation | 22.66 | 1 | 22.66 | 23.46 | < .001 | .09 | [.04, .15] |
| Error | 231.85 | 240 | 0.97 |  |  |  |  |

*Note.* LL and UL represent the lower-limit and upper-limit of the partial η^2^ confidence interval, respectively.

Table S15

*Fixed-Effects ANCOVA results - Conspiracy beliefs about financial crises as the DV*

| Predictor | Sum  of  Squares | *df* | Mean  Square | *F* | *p* | _partial_ η^2^ | _partial_ η^2^  90% CI  [LL, UL] |
| --- | --- | --- | --- | --- | --- | --- | --- |
| (Intercept) | 122.30 | 1 | 122.30 | 69.24 | < .001 |  |  |
| Condition | 37.46 | 2 | 18.73 | 10.60 | < .001 | .08 | [.03, .14] |
| Gender | 0.06 | 1 | 0.06 | 0.04 | .849 | .00 | [.00, .01] |
| Age | 16.52 | 1 | 16.52 | 9.35 | .002 | .04 | [.01, .08] |
| Political orientation | 46.88 | 1 | 46.88 | 26.54 | < .001 | .10 | [.05, .16] |
| Error | 423.91 | 240 | 1.77 |  |  |  |  |

*Note.* LL and UL represent the lower-limit and upper-limit of the partial η^2^ confidence interval, respectively.

Table S16

*Fixed-Effects ANCOVA results - Vaccine-related conspiracy beliefs as the DV*

| Predictor | Sum  of  Squares | *df* | Mean  Square | *F* | *p* | _partial_ η^2^ | _partial_ η^2^  90% CI  [LL, UL] |
| --- | --- | --- | --- | --- | --- | --- | --- |
| (Intercept) | 89.12 | 1 | 89.12 | 32.38 | < .001 |  |  |
| Condition | 116.59 | 2 | 58.30 | 21.18 | < .001 | .15 | [.08, .21] |
| Gender | 2.80 | 1 | 2.80 | 1.02 | .314 | .00 | [.00, .03] |
| Age | 12.34 | 1 | 12.34 | 4.48 | .035 | .02 | [.00, .06] |
| Political orientation | 81.81 | 1 | 81.81 | 29.73 | < .001 | .11 | [.05, .17] |
| Error | 660.53 | 240 | 2.75 |  |  |  |  |

*Note.* LL and UL represent the lower-limit and upper-limit of the partial η^2^ confidence interval, respectively.

**Results of the models with an additional squared term for political orientation**

When we also included quadratic term of political orientation in the ANCOVA, we found a significant main effect of condition, *F*(2, 239) = 14.80, *p* < .001, η_p_² = .11. Post hoc comparisons supported most of our hypotheses. Participants in the low-freedom condition reported significantly higher generic conspiracist beliefs (*M* = 3.63, *SE* = 0.12) than those in the control condition (*M* = 3.13, *SE* = 0.11, *p* = .008, *d* = 0.50) and the high-freedom condition (*M* = 2.75, *SE* = 0.12, *p* < .001, *d* = 0.88). The difference between the control and high-freedom conditions was not significant (*p* = .053, *d* = 0.38), but the effect size was nearly identical to that of the main analysis (*d* = 0.40).

The ANCOVA with included political orientation quadratic term revealed a significant main effect of condition, *F*(2, 239) = 11.47, *p* < .001, η_p_² = .09. Post-hoc comparisons indicated that participants in the low-freedom condition reported significantly higher conspiracy beliefs about financial crises (*M* = 4.74, *SE* = 0.16) compared to those in the high-freedom (*M* = 3.69, *SE* = 0.15, *p* < .001) and control (*M* = 4.19, *SE* = 0.15, *p* = .041) conditions. The difference between the high-freedom and control conditions was not significant (*p* = .061), similarly as in our main analysis (*p* = .085).

For vaccine-related conspiracy beliefs, the analysis with quadratic term of political orientation yielded a significant main effect of condition, *F*(2, 239) = 20.57, *p* < .001, η_p_² = .15. Post-hoc comparisons indicated that participants in the low-freedom condition reported significantly higher vaccine-related conspiracy beliefs (*M* = 4.41, *SE* = 0.20) compared to both the control condition (*M* = 3.67, *SE* = 0.18, *p* = .024) and the high-freedom condition (*M* = 2.66, *SE* = 0.19, *p* < .001). The difference between the control and high-freedom conditions was also statistically significant (*p* < .001).

Table S17

*Fixed-Effects ANCOVA results - Generic conspiracist beliefs as the DV*

| Predictor | Sum  of  Squares | *df* | Mean  Square | *F* | *p* | _partial_ η^2^ | _partial_ η^2^  90% CI  [LL, UL] |
| --- | --- | --- | --- | --- | --- | --- | --- |
| (Intercept) | 52.10 | 1 | 52.10 | 53.84 | < .001 |  |  |
| Condition | 28.63 | 2 | 14.31 | 14.80 | < .001 | .11 | [.05, .17] |
| Gender | 0.03 | 1 | 0.03 | 0.03 | .853 | .00 | [.00, .01] |
| Age | 0.17 | 1 | 0.17 | 0.17 | .680 | .00 | [.00, .02] |
| Political orientation | 0.15 | 1 | 0.15 | 0.15 | .697 | .00 | [.00, .02] |
| Political orientation squared | 0.60 | 1 | 0.60 | 0.62 | .432 | .00 | [.00, .02] |
| Error | 231.25 | 239 | 0.97 |  |  |  |  |

*Note.* LL and UL represent the lower-limit and upper-limit of the partial η^2^ confidence interval, respectively.

Table S18

*Fixed-Effects ANCOVA results - Conspiracy beliefs about financial crises as the DV*

| Predictor | Sum  of  Squares | *df* | Mean  Square | *F* | *p* | _partial_ η^2^ | _partial_ η^2^  90% CI  [LL, UL] |
| --- | --- | --- | --- | --- | --- | --- | --- |
| (Intercept) | 78.00 | 1 | 78.00 | 44.67 | < .001 |  |  |
| Condition | 40.05 | 2 | 20.02 | 11.47 | < .001 | .09 | [.04, .14] |
| Gender | 0.04 | 1 | 0.04 | 0.02 | .879 | .00 | [.00, .01] |
| Age | 15.56 | 1 | 15.56 | 8.91 | .003 | .04 | [.01, .08] |
| Political orientation | 0.76 | 1 | 0.76 | 0.43 | .511 | .00 | [.00, .02] |
| Political orientation squared | 6.63 | 1 | 6.63 | 3.80 | .052 | .02 | [.00, .05] |
| Error | 417.27 | 239 | 1.75 |  |  |  |  |

*Note.* LL and UL represent the lower-limit and upper-limit of the partial η^2^ confidence interval, respectively.

Table S19

*Fixed-Effects ANCOVA results - Vaccine-related conspiracy beliefs as the DV*

| Predictor | Sum  of  Squares | *df* | Mean  Square | *F* | *p* | _partial_ η^2^ | _partial_ η^2^  90% CI  [LL, UL] |
| --- | --- | --- | --- | --- | --- | --- | --- |
| (Intercept) | 21.78 | 1 | 21.78 | 7.91 | .005 |  |  |
| Condition | 113.33 | 2 | 56.66 | 20.57 | < .001 | .15 | [.08, .21] |
| Gender | 2.70 | 1 | 2.70 | 0.98 | .323 | .00 | [.00, .03] |
| Age | 12.78 | 1 | 12.78 | 4.64 | .032 | .02 | [.00, .06] |
| Political orientation | 12.66 | 1 | 12.66 | 4.59 | .033 | .02 | [.00, .06] |
| Political orientation squared | 2.08 | 1 | 2.08 | 0.76 | .385 | .00 | [.00, .03] |
| Error | 658.45 | 239 | 2.76 |  |  |  |  |

*Note.* LL and UL represent the lower-limit and upper-limit of the partial η^2^ confidence interval, respectively.

**Supplementary analyses on data from Study 5**

Table S20

*Means, standard deviations, and correlations for variables used in Study 5*

| Variable | *M* | *SD* | 1 | 2 | 3 | 4 | 5 | 6 | 7 | 8 | 9 |
| --- | --- | --- | --- | --- | --- | --- | --- | --- | --- | --- | --- |
|  |  |  |  |  |  |  |  |  |  |  |  |
| 1. Perceived societal freedom | 5.22 | 1.13 |  |  |  |  |  |  |  |  |  |
|  |  |  |  |  |  |  |  |  |  |  |  |
| 2. Generic conspiracy beliefs | 4.65 | 1.69 | -.11** |  |  |  |  |  |  |  |  |
|  |  |  |  |  |  |  |  |  |  |  |  |
| 3. Generic conspiracist beliefs | 3.10 | 1.27 | -.14*** | .70*** |  |  |  |  |  |  |  |
|  |  |  |  |  |  |  |  |  |  |  |  |
| 4. Vaccine-related conspiracy beliefs | 3.64 | 1.96 | -.08 | .65*** | .63*** |  |  |  |  |  |  |
|  |  |  |  |  |  |  |  |  |  |  |  |
| 5. Conspiracy beliefs about financial crises | 4.47 | 1.59 | -.04 | .67*** | .72*** | .62*** |  |  |  |  |  |
|  |  |  |  |  |  |  |  |  |  |  |  |
| 6. Lack of control | 3.51 | 1.14 | -.24*** | .08 | .12** | .04 | .07 |  |  |  |  |
|  |  |  |  |  |  |  |  |  |  |  |  |
| 7. Political anger | 3.77 | 1.65 | -.09* | .20*** | .17*** | .15*** | .20*** | .19*** |  |  |  |
|  |  |  |  |  |  |  |  |  |  |  |  |
| 8. Gender | 0.47 | 0.50 | -.01 | -.01 | .01 | -.10* | .0003 | -.04 | .05 |  |  |
|  |  |  |  |  |  |  |  |  |  |  |  |
| 9. Age | 47.74 | 16.40 | .28*** | .001 | -.002 | .07 | .11* | -.14*** | .14*** | -.07 |  |
|  |  |  |  |  |  |  |  |  |  |  |  |
| 10. Political orientation | 4.07 | 1.60 | .06 | .35*** | .28*** | .39*** | .28*** | -.01 | .11** | .09* | -.003 |
|  |  |  |  |  |  |  |  |  |  |  |  |

*Note.* * indicates *p* < .05. ** indicates *p* < .01. *** indicates *p* < .001. Gender was a dichotomous variable with values of 0 = woman and 1 = man.

**Results of the models with an additional squared term for political orientation**

Across all models, perceived societal freedom significantly predicted both mediators. It was associated with a lower sense of lack of control (*B* = -0.23, *SE* = 0.04, *p* < .001) and lower levels of political anger (*B* = -0.24, *SE* = 0.06, *p* < .001). Among the covariates, age was negatively associated with a lack of control (*B* = -0.01, *SE* = 0.003, *p* = .048) and positively associated with political anger (*B* = 0.02, *SE* = 0.004, *p* < .001). Gender did not have a significant effect on lack of control (*B* = -0.12, *SE* = 0.09, *p* = .177) and political anger (*B* = 0.12, *SE* = 0.13, *p* = .384). Political orientation was not significantly associated with a lack of control (*B* = -0.10, *SE* = 0.13, *p* = .455), but it was negatively related to political anger (*B* = -0.61, *SE* = 0.19, *p* = .001). The squared term of political orientation was positively associated with political anger (*B* = 0.09, *SE* = 0.02, *p* < .001), but not with lack of control (*B* = 0.01, *SE* = 0.02, *p* = .418).

***Generic conspiracy beliefs***

The analysis revealed that political anger positively predicted generic conspiracy beliefs (*B* = 0.16, *SE* = 0.04, *p* < .001). However, the effect of lack of control was not significant (*B* = 0.04, *SE* = 0.06, *p* = .510). The indirect effect of perceived societal freedom via political anger (*IE* = -0.04, *SE_boot_* = 0.02, 95% *CI* [-0.07, -0.01]) was significant, while the indirect effect through lack of control was not significant (*IE* = -0.01, *SE_boot_* = 0.01, 95% *CI* [-0.04, 0.02]). After accounting for the effects of the mediators, the direct effect of perceived societal freedom on generic conspiracy beliefs was significant (*B* = -0.17, *SE* = 0.06, *p* = .006), similarly to the total effect (*B* = -0.22, β = -.14, *SE* = 0.06, *p* < .001), which indicates a partial mediation. Among the covariates, age had non-significant effect (*B* = 0.001, *SE* = 0.004, *p* = .729) on generic conspiracy beliefs, similarly as gender (*B* = -0.15, *SE* = 0.13, *p* = .251), political orientation (*B* = 0.35, *SE* = 0.18, *p* = .054), and political orientation squared term (*B* = 0.001, *SE* = 0.02, *p* = .953). Figure S1 presents the summary of the model with standardized coefficients.

Figure S1

*The effect of perceived societal freedom on generic conspiracy beliefs via lack of control and political anger*


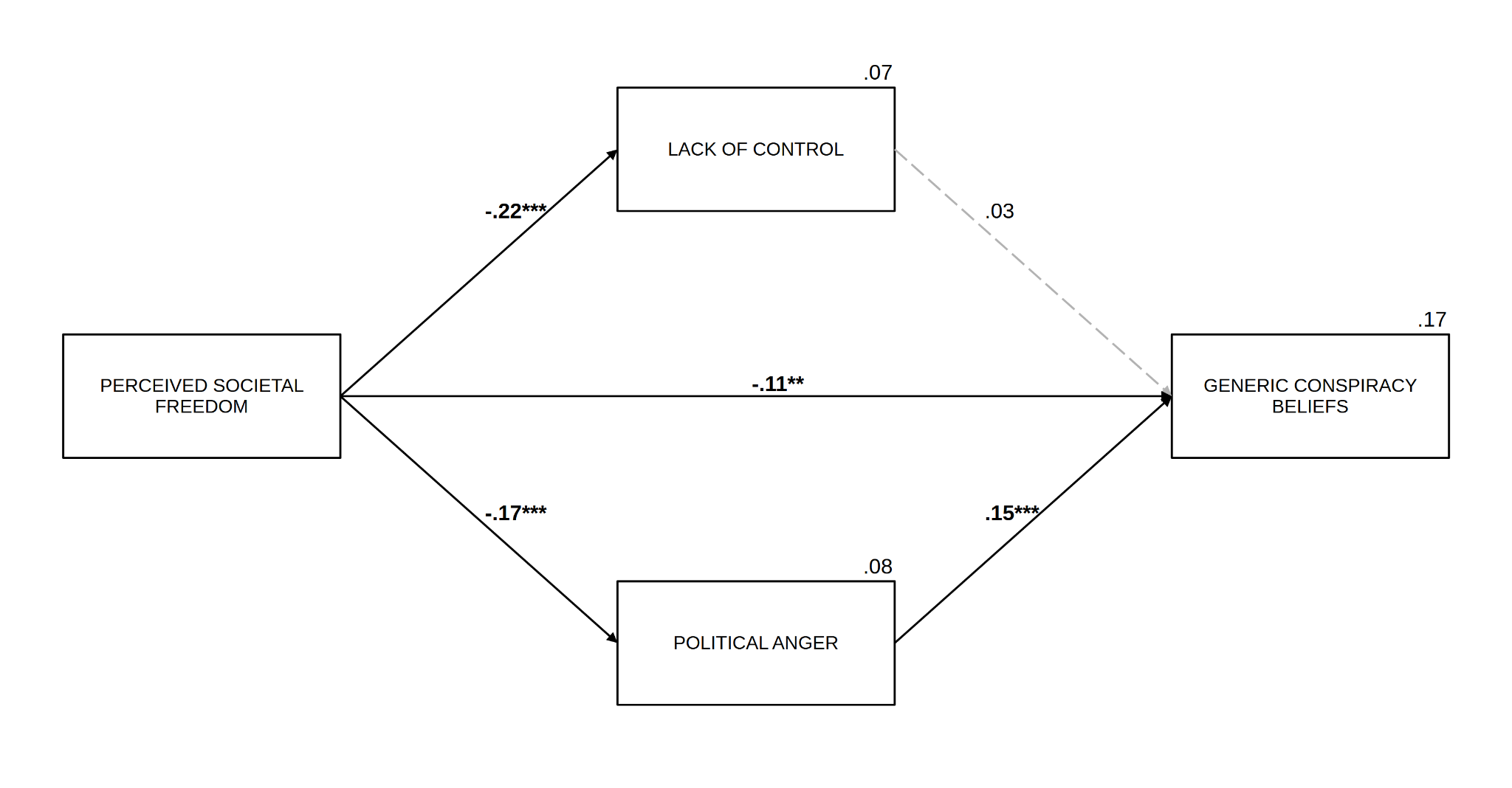


*Note*. ** *p* < .01. *** *p* < .001. The figure presents standardized coefficients. Dashed lines represent non-significant effects. The value between the main predictor and the dependent variable indicates the direct effect. We controlled for age, gender, and

political orientation in the analysis.

***Generic conspiracist beliefs***

Political anger emerged as a significant predictor of generic conspiracist beliefs (*B* = 0.09, *SE* = 0.03, *p* = .005), while the effect of lack of control (*B* = 0.07, *SE* = 0.05, *p* = .102) was non-significant. The indirect effect via political anger (*IE* = -0.02, *SE_boot_* = 0.01, 95% *CI* [-0.05, -0.004]) was significant, while the one through and lack of control was non-significant (*IE* = -0.02, *SE_boot_* = 0.01, 95% *CI* [-0.04, 0.004]). After controlling for mediators, perceived societal freedom maintained a significant direct effect on generic conspiracist beliefs (*B* = -0.16, *SE* = 0.05, *p* < .001). The total effect was also significant (*B* = -0.20, β = -.17, *SE* = 0.05, *p* < .001). Among the covariates, age showed no significant effect (*B* = 0.002, *SE* = 0.003, *p* = .458), similarly as gender (*B* = -0.03, *SE* = 0.10, *p* = .775), political orientation (*B* = 0.26, *SE* = 0.14, *p* = .063), and the squared term of political orientation (*B* = -0.005, *SE* = 0.02, *p* = .785). See Figure S2 for a summary of the model with standardized coefficients.

Figure S2

*The effect of perceived societal freedom on generic conspiracist beliefs via lack of control and political anger*


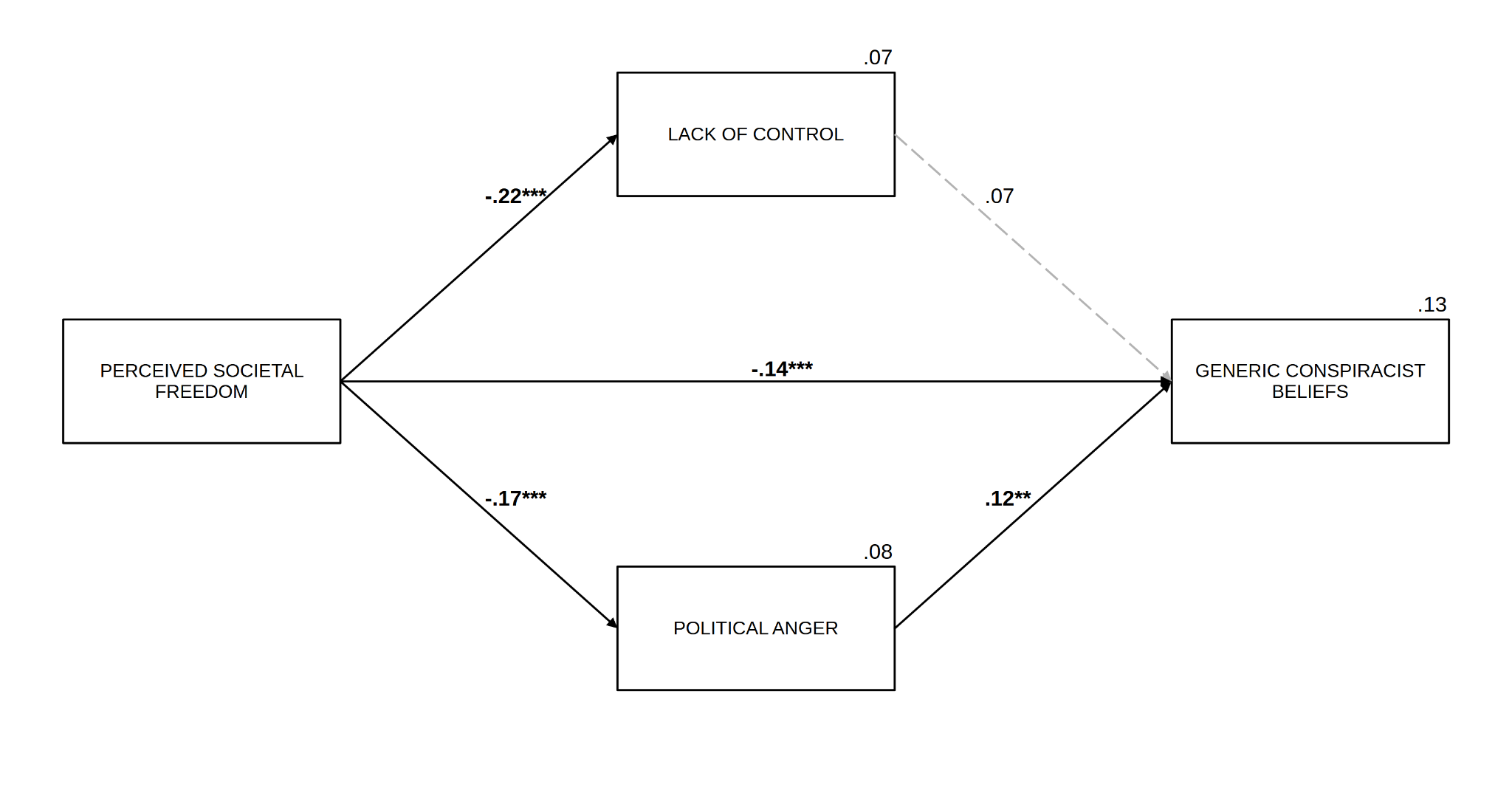


*Note*. ** *p* < .01. *** *p* < .001. The figure presents standardized coefficients. Dashed lines represent non-significant effects. The value between the main predictor and the dependent variable indicates the direct effect. We controlled for age, gender, and

political orientation in the analysis.

***Vaccine-related conspiracy beliefs***

In the case of vaccine-related conspiracy beliefs, political anger (*B* = 0.11, *SE* = 0.05, *p* = .024) was a significant predictor, while the effect of lack of control (*B* = 0.002, *SE* = 0.07, *p* = .975) was not significant. Consistent with this, indirect effect via political anger (*IE* = -0.03, *SE_boot_* = 0.01, 95% *CI* [-0.06, -0.002]) was significant and the one through and lack of control (*IE* = -0.001, *SE_boot_* = 0.02, 95% *CI* [-0.3, 0.03]) was not significant. The direct effect (*B* = -0.21, *SE* = 0.07, *p* = .003) remained significant after accounting the effects of mediators, as was the total effect (*B* = -0.23, β = -.13, *SE* = 0.07, *p* < .001). In this model, age positively predicted vaccine-related conspiracy beliefs (*B* = 0.01, *SE* = 0.005, *p* = .035). Gender had a significant negative effect (*B* = -0.53, *SE* = 0.15, *p* < .001) on this variable, while the effect of political orientation (*B* = 0.43, *SE* = 0.21, *p* = .037) was positive. However, the effect of political orientation squared term (*B* = 0.01, *SE* = 0.02, *p* = .759) was not significant. Figure S3 shows a summary of the model with standardized coefficients.

Figure S3

*The effect of perceived societal freedom on vaccine-related conspiracy beliefs via lack of control and political anger*


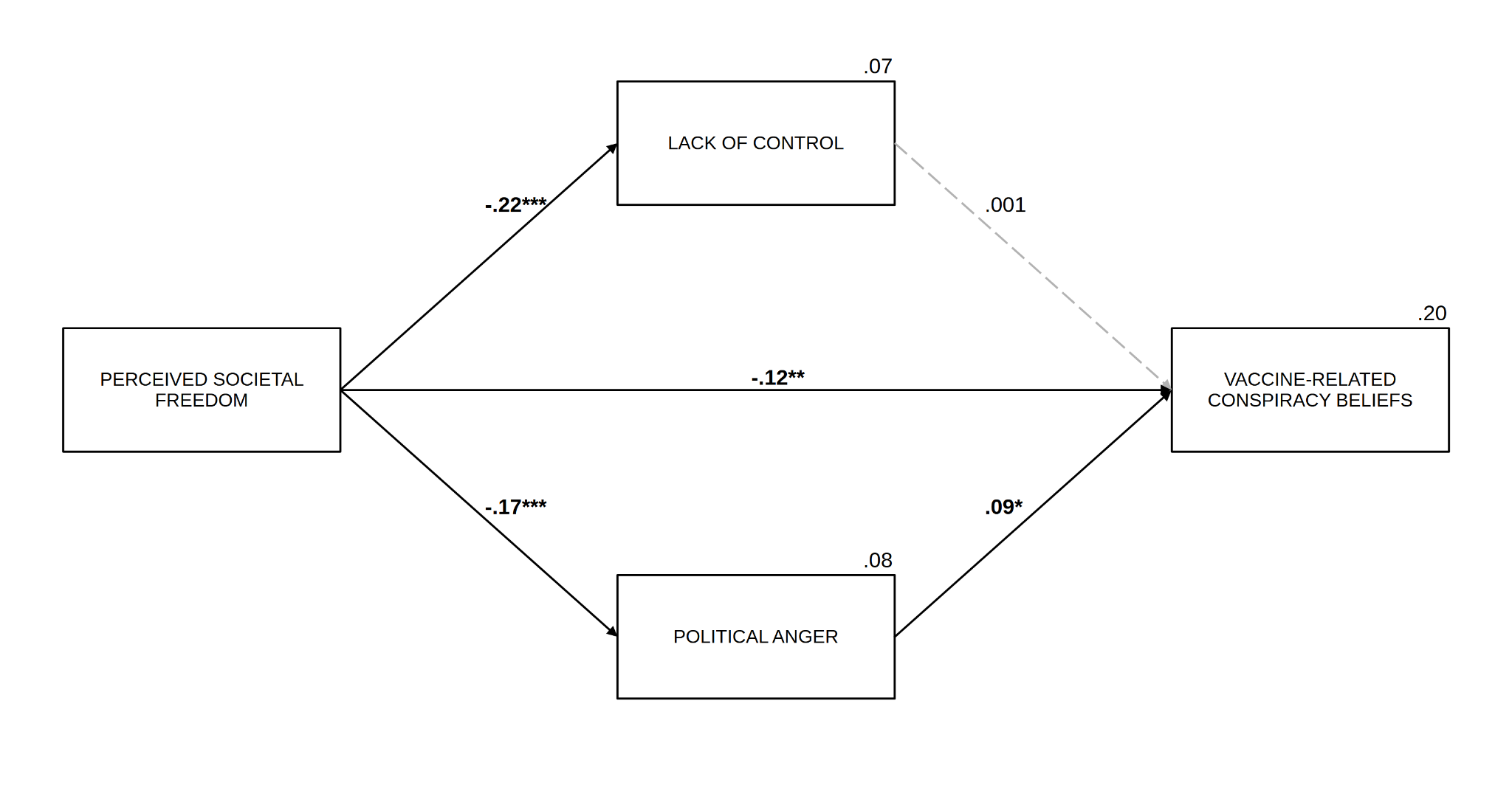


*Note*. * *p* < .05. ** *p* < .01. *** *p* < .001. The figure presents standardized coefficients. Dashed lines represent non-significant effects. The value between the main predictor and the dependent variable indicates the direct effect. We controlled for age, gender, and

political orientation in the analysis.

***Conspiracy beliefs about financial crises***

Political anger was a significant predictor of conspiracy beliefs about financial crises (*B* = 0.13, *SE* = 0.04, *p* = .001). However, the effect of lack of control was not significant (*B* = 0.07, *SE* = 0.06, *p* = .239). This pattern was reflected in a significant indirect effect through political anger (*IE* = -0.03, *SE_boot_* = 0.01, 95% *CI* [-0.06, -0.01]). In contrast, the indirect effect through the lack of control was not significant (*IE* = -0.02, *SE_boot_* = 0.01, 95% *CI* [-0.05, 0.01]). The direct effect was not significant this time (*B* = -0.10, *SE* = 0.06, *p* = .102), while the total effect was significant (*B* = -0.14, β = -.10, *SE* = 0.06, *p* = .014), suggesting a complete mediation. Among the covariates, age (*B* = 0.01, *SE* = 0.004, *p* = .007) positively predicted conspiracy beliefs about financial crises, while the effects of gender (*B* = -0.08, *SE* = 0.12, *p* = .528), political orientation (*B* = 0.10, *SE* = 0.18, *p* = .568), and the squared term of political orientation (*B* = 0.02, *SE* = 0.02, *p* = .308) were not significant. For a summary of the model with standardized coefficients, see Figure S4.

Figure S4

*The effect of perceived societal freedom on conspiracy beliefs about financial crises via lack of control and political anger*


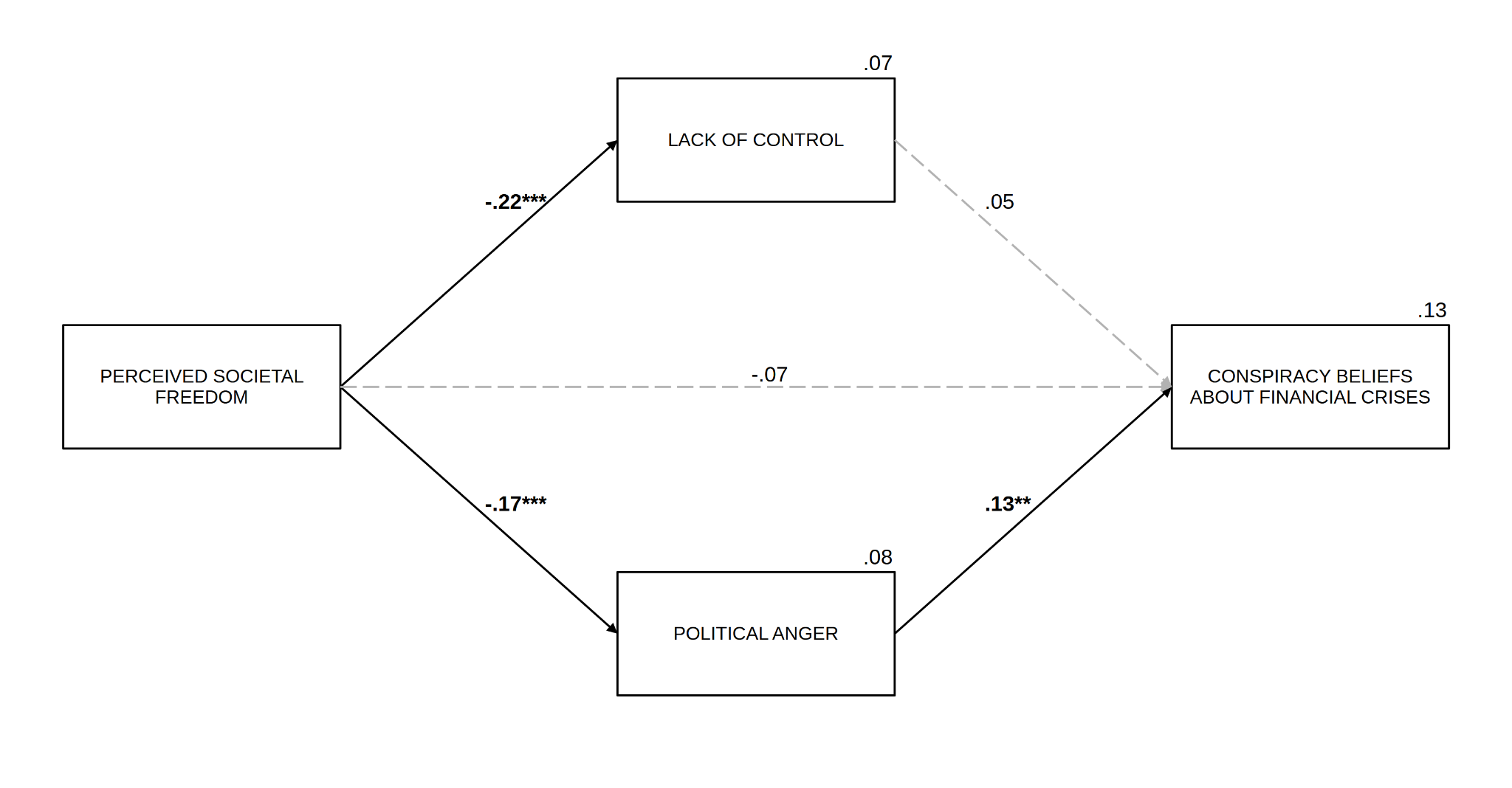


*Note*. ** *p* < .01. *** *p* < .001. The figure presents standardized coefficients. Dashed lines represent non-significant effects. The value between the main predictor and the dependent variable indicates the direct effect. We controlled for age, gender, and

political orientation in the analysis.
